# A computational approach for the discovery of significant cancer genes by weighted mutation and asymmetric spreading strength in networks

**DOI:** 10.1038/s41598-021-02671-8

**Published:** 2021-12-07

**Authors:** Jorge Francisco Cutigi, Adriane Feijo Evangelista, Rui Manuel Reis, Adenilso Simao

**Affiliations:** 1 Federal Institute of Sao Paulo, Sao Carlos, SP Brazil; 2 Molecular Oncology Research Center, Barretos Cancer Hospital, Barretos, SP Brazil; 3 University of Sao Paulo, Sao Carlos, SP Brazil

**Keywords:** Computational biology and bioinformatics, Cancer genomics

## Abstract

Identifying significantly mutated genes in cancer is essential for understanding the mechanisms of tumor initiation and progression. This task is a key challenge since large-scale genomic studies have reported an endless number of genes mutated at a shallow frequency. Towards uncovering infrequently mutated genes, gene interaction networks combined with mutation data have been explored. This work proposes Discovering Significant Cancer Genes (DiSCaGe), a computational method for discovering significant genes for cancer. DiSCaGe computes a mutation score for the genes based on the type of mutations they have. The influence received for their neighbors in the network is also considered and obtained through an asymmetric spreading strength applied to a consensus gene network. DiSCaGe produces a ranking of prioritized possible cancer genes. An experimental evaluation with six types of cancer revealed the potential of DiSCaGe for discovering known and possible novel significant cancer genes.

## Introduction

Cancer cells undergo a large number of somatic mutations, of which most are passenger mutations, i.e., typically random mutations that do not contribute to carcinogenesis^1^. A critical issue in cancer genomics is identifying a small group of significant mutations for cancer, i.e., those responsible for cancer initiation and progression, called driver mutations (a gene containing driver mutations can be called driver gene or significant gene). Such a task is challenging since cancer is characterized by a high heterogeneity of genetic mutations, i.e., a small number of genes is mutated at high frequency in a cohort of patients, and a high number of genes is low-frequency mutated^2,3^. This phenomenon is known as “long tail”, and is related to an intrinsic difficulty in identifying commonly mutated genes through their mutation frequency. Some mutated genes in the tail (at low mutation frequency) can have an important biological role in tumorigenesis, thus posing a statistical difficulty since it is not enough to mention that genes with the highest frequency of mutation are drivers. The advent of next-generation sequencing technologies (NGS) has generated a large volume of biological data in a short time, which are subsidies for several computational methods for the identification of significant mutations in cancer genes^4–8^.

Computational methods have adopted several strategies to overcome this issue, such as gene interaction networks, used to study mutated gene’s interactions and their influence on networks^9–13^. Network analysis is essential, since genes affected by driver mutations tend to participate in common biological activities^14^. Furthermore, somatic mutations can alter gene function and, therefore, its entire pathways^15^.

This manuscript introduces DiSCaGe (*Di*scovering *S*ignificant *Ca*ncer *Ge*nes), a computational method for prioritizing significant genes for cancer. Such significance is directly related to the impact of different mutation types and gene interactions on networks. DiSCaGe is based on the hypothesis that genes involved in cancer tend to interact with each other^16^, and the mutations they undergo can influence their neighborhood. This influence is extracted from asymmetric spreading strength measures of all node pairs, which take into account direct and indirect neighbors on the network. Such spreading strength is used to quantify how much its neighborhood’s mutated genes can perturb a gene.

DiSCaGe prioritized mutated genes in six types of cancer: breast invasive carcinoma (BRCA), colorectal adenocarcinoma (COADREAD), glioblastoma multiforme (GBM), lung adenocarcinoma, prostate adenocarcinoma (PRAD), and stomach adenocarcinoma (STAD). The method uses mutation data sets and two gene interaction networks: Reactome Functional Interactions (ReactomeFI) and Human Protein Reference Database (HPRD). Cancer mutation data were subjected to a preprocessing routine, and networks underwent a link prediction process. Lists of prioritized genes were evaluated concerning precision and discounted cumulative gain, using recent cancer driver genes benchmarks. The results showed DiSCaGe’s potential for discovering known and possible novel cancer genes, including very-low-frequency mutated genes.

## Results

### DiSCaGe overview

**Figure 1:**
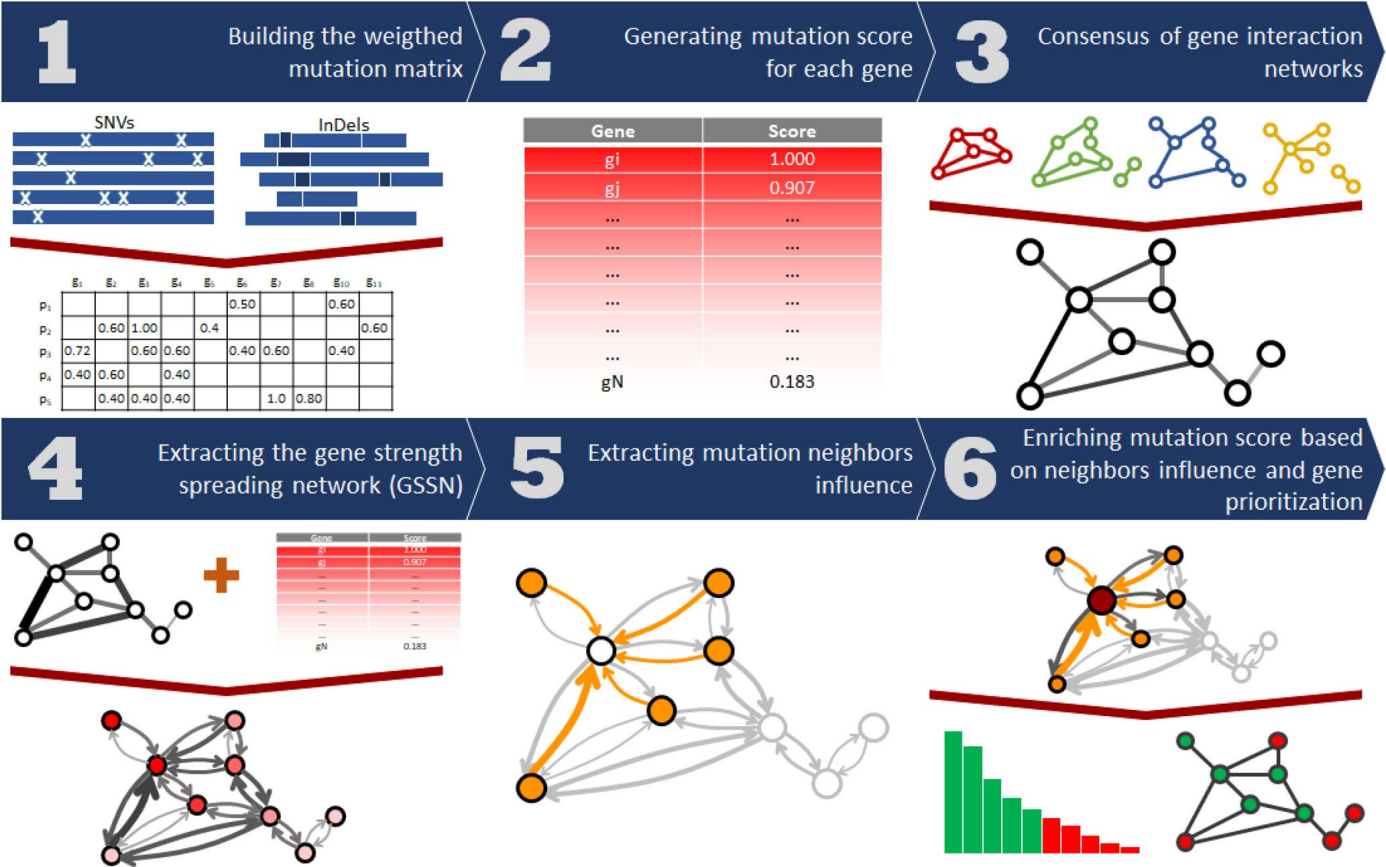
DiSCaGe overview.

DiSCaGe uses cancer mutation data (SNVs and InDels in an MAF file format) and a set of $N \ge 1$ undirected and unweighted gene interaction networks (in edge lists) as input, whereas the output is a ranking of prioritized mutated cancer genes. DiSCaGe is composed of 6 steps, as illustrated in Fig. 1. In Step 1, a weighted mutation matrix (WMM) is built and assigned a real value for each patient-gene pair, according to the weight defined for the variant classification of the mutation and the number of mutated patients. Step 2 uses WMM to obtain a mutation score for each gene, called weighted mutation frequency. Next, in Step 3, a union operation is performed on the gene interactions networks, resulting in an undirected and weighted consensus gene interaction network. In Step 4, a gene spreading strength network (GSSN) is obtained, according to the spreading strength from a gene to its direct and indirect neighbors. Step 5 extracts a mutation influence exerted on all genes by their neighbors, based on GSSN and gene mutation scores. Finally, in Step 6, each gene mutation score is enriched with the neighbors’ influence, and a sorted list of prioritized genes is obtained. All the steps are described in the Methods section. The algorithms and a running example can be found in the supplementary material.

### Experimental study

The following six cancer mutation data sets were selected for the experiments that aimed at finding significant mutations. Such types are among the most common types of cancer, according to World Health Organization^17^, and GBM commonly appears in method’s evaluation in several studies: (1) Breast invasive carcinoma (BRCA); (2) Colorectal adenocarcinoma (COADREAD); (3) Glioblastoma multiforme (GBM); (4) Lung adenocarcinoma (LUAD); (5) Prostate adenocarcinoma (PRAD); and (6) Stomach adenocarcinoma (STAD). MAF files (Mutation Annotation Format) with Single Nucleotide Variants (SNVs) and Insertions and Deletions (InDels) were extracted from a Pan-Cancer study of TCGA^3^ through cBioPortal (https://www.cbioportal.org/datasets), which is an interactive platform for the exploration of cancer data^18,19^. Mutation weights used in the experiments are presented in the supplementary material. The enriched versions of the following two gene interaction networks were used on DiSCaGe execution: (1) Reactome Functional Interactions (ReactomeFI)^20–22^; and (2) Human Protein Reference Database (HPRD)^23,24^. The selected networks were subjected to an enrichment process towards inferences about possible new interactions among the genes (see Gene interaction networks for further details).

#### Evaluation metrics

The evaluation of computational methods that identify significant mutations in cancer remains a challenging task. The lack of gold-standard databases for driver and passenger genes hampers obtaining an optimal measure of the output. Nonetheless, some gene databases are widely used and continuously updated. Towards a well-defined set of known cancer genes, the following six reliable and recent available benchmarks were considered:

1. A $D_{NCG}$ set of 711 known cancer drivers extracted from the Network of Cancer Genes (NCG) (Version 6.0—http://ncg.kcl.ac.uk/download.php)^25^.
2. A $D_{CGC}$ set of 723 driver genes extracted from Cancer Gene Census (CGC) (Version 92, 27-Aug-20—https://cancer.sanger.ac.uk/census)^26,27^.
3. A $D_{IntOGen}$ set of 568 driver genes extracted from Integrative OncoGenomics (IntOGen) (Release 2020-02-01—https://www.intogen.org/search)^28^.
4. A $D_{Bailey}$ set of 299 driver genes extracted from the recent and extensive study conducted by^3^ (Baylei et al. 2018—https://pubmed.ncbi.nlm.nih.gov/29625053/).
5. An $FD_{NCG}$ set of 250 genes listed as possible false positive drivers (Version 6.0—http://ncg.kcl.ac.uk/false_positives.php) by the Network of Cancer Genes (NCG).
6. Six $SD^{cancer\_type}_{IntOGen}$ sets of specific drivers for each type of cancer, based on the known specific driver from IntOGen, i.e., each set contains genes related to a specific type of cancer, whose specific benchmark is essential, since many genes are important in specific types of cancer, and probably irrelevant in others^29^. The sets $SD^{BRCA}_{IntOGen}$, $SD^{COADREAD}_{IntOGen}$, $SD^{GBM}_{IntOGen}$, $SD^{LUAD}_{IntOGen}$, $SD^{PRAD}_{IntOGen}$, and $SD^{STAD}_{IntOGen}$ have 99, 72, 35, 42, 82 and 61 specific drivers, respectively.

With these sets of known driver gene benchmarks, the following metrics were used to evaluate the proposed approach:

- *Precision:* the fraction of prioritized genes that are known to be related to cancer. The precision of the ranking can be computed and is obtained by $Precision~=~\frac{|PG \cap D|}{|PG|}$, where *PG* is the set of prioritized genes, and *D* is the set of known driver genes. A union of the four described benchmarks was performed to obtain set *D*, resulting in a single set of 951 known cancer drivers, i.e., $D = D_{NCG} \cup D_{CGC} \cup D_{IntOGen} \cup D_{Bailey}$.
- *Discounted Cumulative Gain (DCG):* a measure that considers the relevance of the genes and their position in the ranking, being reduced logarithmically, proportional to this position^30^. $DCG_p$ of a ranking of genes up to position *p* is defined as $DCG_{p} = \sum _{i=1}^{PG_p}{\frac{rel^{ct_j}_{g_i}}{\log _{2}(i+1)}}$, where $PG_p$ is the ranking list of *p* prioritized genes and $rel^{ct_j}_{g_i}$ is the relevance of a gene $g_i$ in cancer type $ct_j$. Such relevance is calculated by incrementing 1 whenever $g_i$ is contained in $D_{NCG}$, $D_{CGC}$, $D_{IntOGen}$, or $D_{Bailey}$. If $g_i$ is contained in specific driver benchmark $SD_{IntOGen}^{ct_j}$, then $rel^{ct_j}_{g_i}$ is incremented by 4 (thus, specific cancer drivers have the same weight if it appears in all four general benchmarks), if it is contained in $FD_{NCG}$, then $rel^{ct_j}_{g_i}$ is decreased by 1. Finally, if $g_i$ is not in any gene set, its value is zero.

#### Top 30 genes on benchmarks

**Figure 2:**
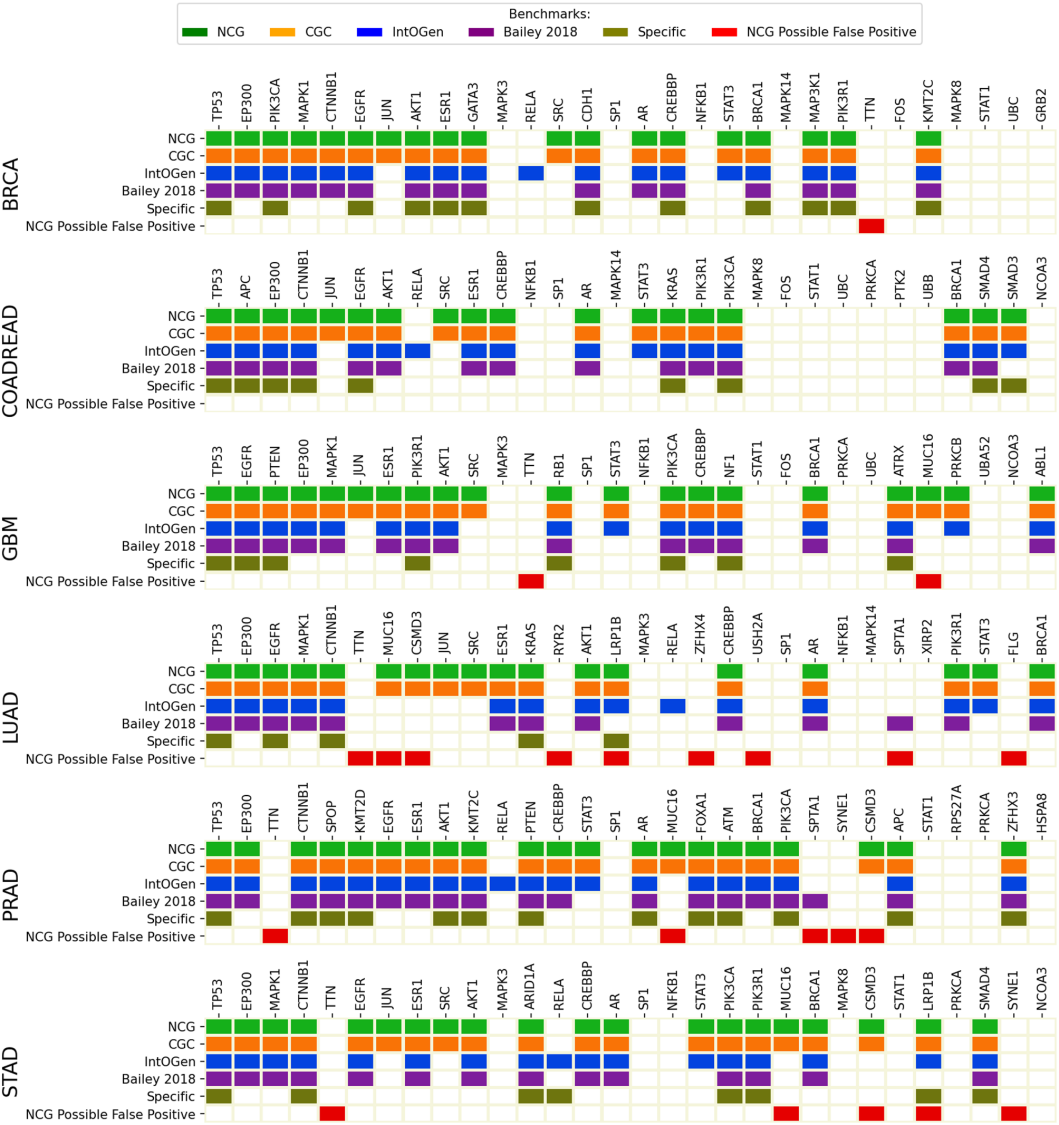
How top 30 genes appear in benchmarks.

The top 30 prioritized genes for each type of cancer were selected to be presented in a matrix for each type, that shows the presence of prioritized genes on the benchmarks, as seen in Fig. 2. Each driver gene benchmark is presented in a row with a specific color, and genes discovered by DiSCaGe are presented in the columns; for example, NCG is the first row and has the green color. If a gene is contained in the benchmark, the matrix cell is colored; otherwise, it is white. It can be noticed how the genes are arranged on the benchmarks and show that results are consistent. Some important genes appear in the results, which are reported on most benchmarks. Few genes do not appear in any benchmark, which can be subjected to further analysis.

#### Comparison with related methods

In this experiment, DiSCaGe is compared with related methods MutSigCV^31^, MUFFINN^10^, and nCOP^11^, which were employed with standard parameters and configurations towards avoiding possible running influence, described as follows: (1) MutSigCV: executed with only MAF file. A difference in the preprocessing routine was assigned to MAF files for MutSigCV, in which Silent mutations were kept in MAF, as they are mandatory data for the method, which uses them to extract the background mutation rate; (2) MUFFIN: executed with the number of mutated patients for each gene as the gene mutation score (mutation occurrence data). MUFFINN has four variations, which are a combination of the approach (DNmax or DNsum) with the gene network (STRING or HumanNet). DNmax + HumanNet was selected for this experiment because it provided the best results; and (3) nCOP: executed with its preprocessed HPRD gene network with no weights for the nodes in the network. The optimal value for alpha was obtained for nCOP itself.

**Figure 3:**
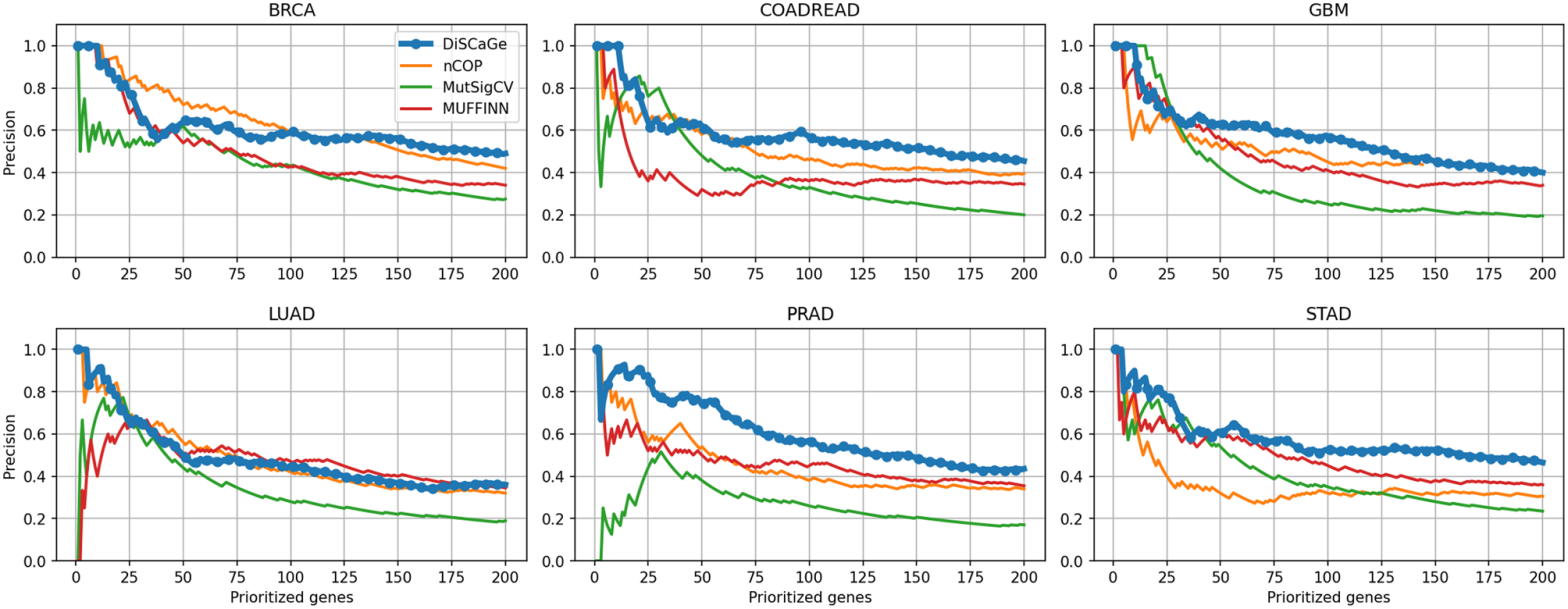
Precision of DiSCaGe and related methods: shows the fraction of prioritized genes (y-axis) contained in the known driver benchmarks for a specific number of genes (x-axis).

**Figure 4:**
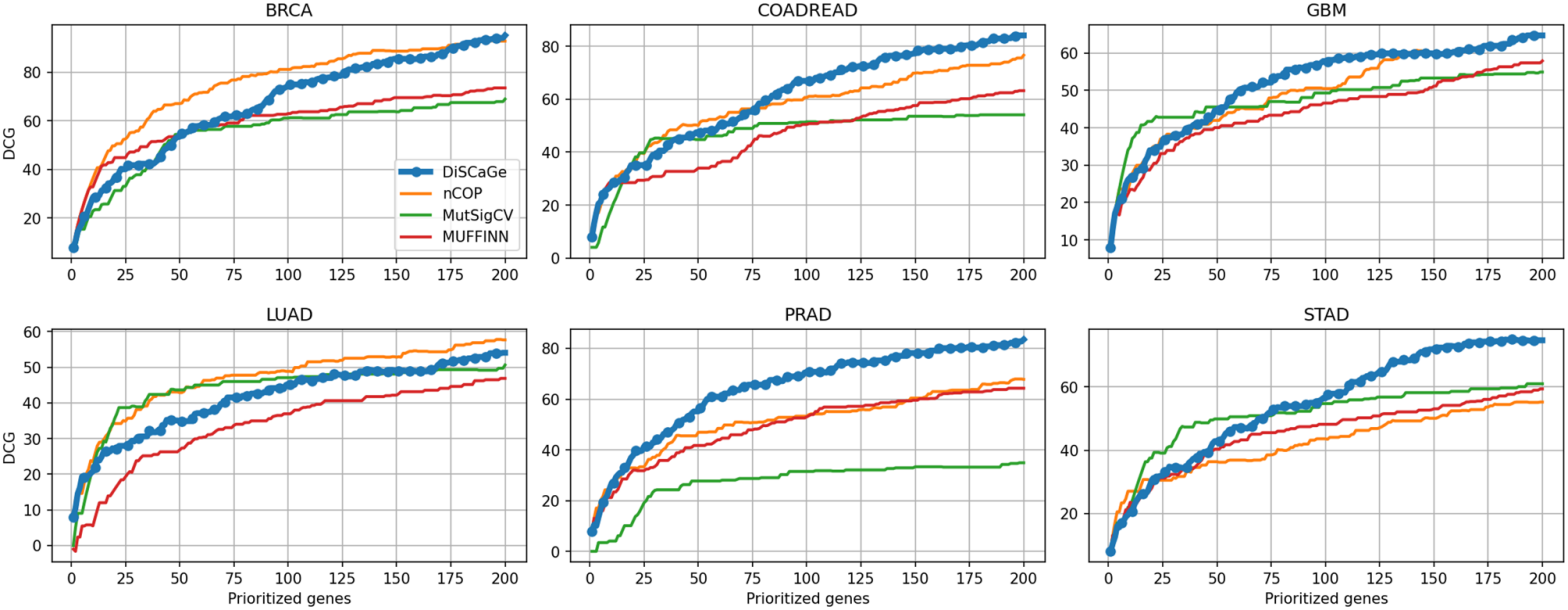
DCG of DiSCaGe and related methods: the relevance (y-axis) of a ranking of a specific number of genes (x-axis), taking into account their position in the ranking and their appearance in the driver benchmarks.

Figures 3 and 4 display precision and DCG, respectively, for all methods. The results show variations according to the type of cancer and the top *N* value. For example, considering precision, for BRCA, nCOP is better for *N* nearly from 15 to 100. In general, DiSCaGe outperformed all methods for most values of *N* for COADREAD, GBM, PRAD, and STAD. DCG showed a higher variation in the results. Although DiSCaGe outperformed all methods for PRAD, this performance was neither dominant nor explicit for the other types of cancer. nCOP is clearly better for BRCA, and MutSigCV yielded promising results for STAD and GBM up to *N* nearly 60. DiSCaGe outperformed all methods for COADREAD, GBM, and STAD for *N* larger than nearly 75.

#### Prioritization of low-frequency mutated genes

**Figure 5:**
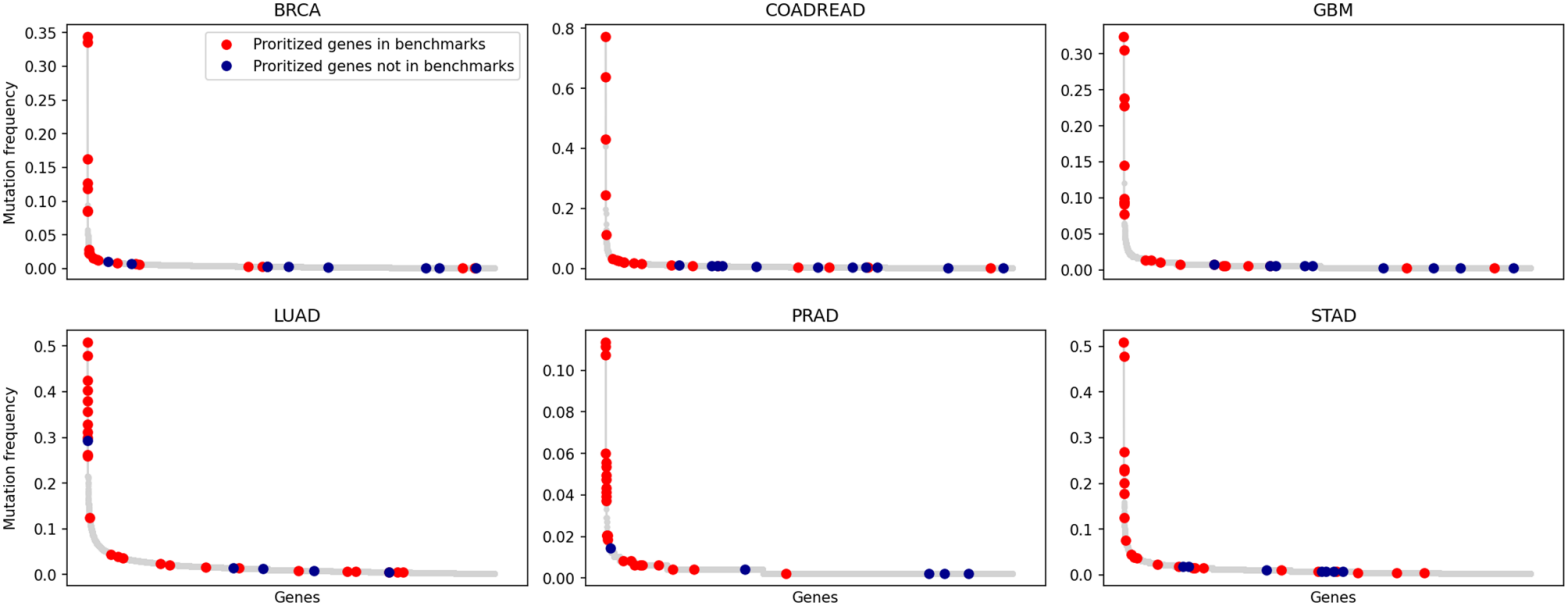
Low-frequency mutated genes.

DiSCaGe can find low-frequency cancer mutated genes. Figure 5 shows the long-tail chart for each cancer data set studied. The top 30 prioritized genes are highlighted in the charts, where red dots are genes known to be related to cancer, and blue ones are possible cancer genes prioritized by DiSCaGe. The gene names can be observed in the matrix of Fig. 2, in which the blue genes are not contained in any benchmark, i.e., the matrix column is composed of only white cells. Several prioritized genes are on the tail of the graph, thus showing the potential of DiSCaGe for prioritizing known and low-frequency cancer genes and possible novel ones.

#### Automated literature-based analysis and in silico validation

**Figure 6:**
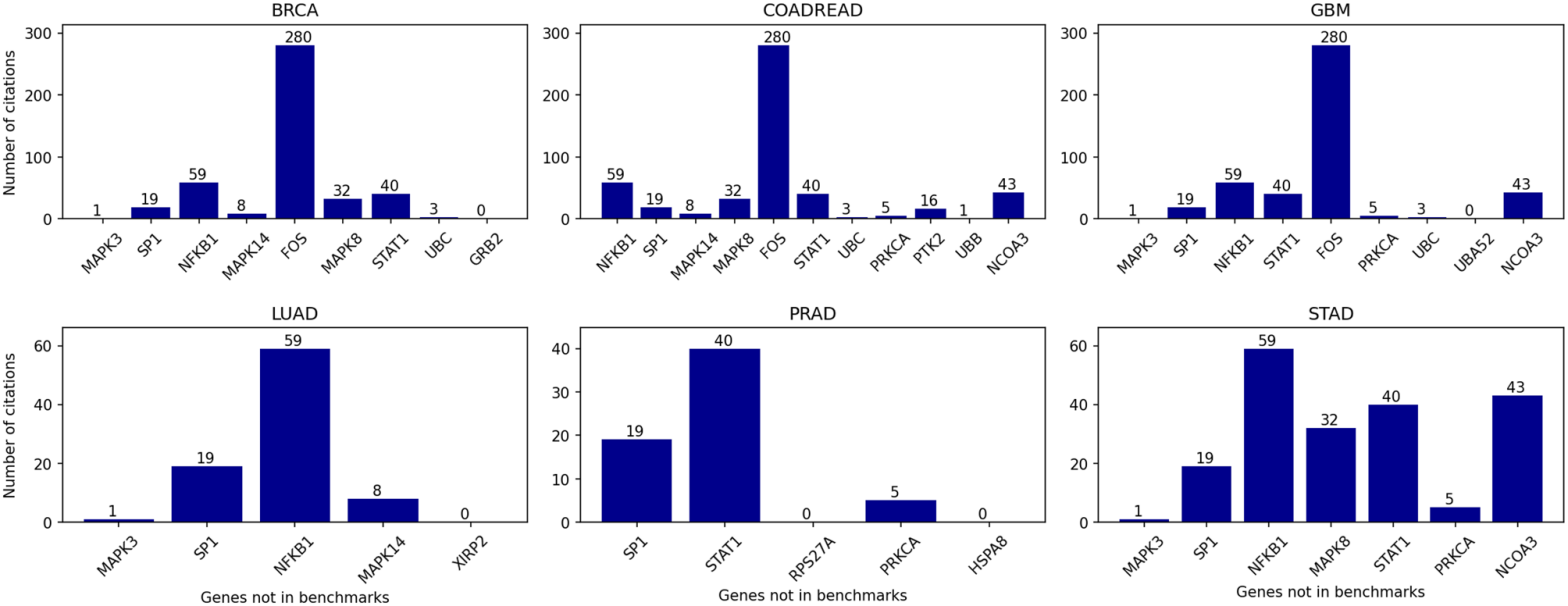
A secondary study for the DiSCaGe results: Number of papers (y-axis), reported by CancerMine, that mention specific genes (x-axis) as “*cancer gene*”. DiSCaGe prioritized the presented genes, and they are not contained in any known driver gene benchmark.

As shown in Figs. 2 and 5, some genes are not in any driver gene benchmarks. Their prioritization suggests they can potentially be novel cancer genes. Towards a secondary study on those genes, an automated literature review was performed using CancerMine^29^, a recent tool based on text mining. CancerMine provides information on genes in the cancer context, classifying them as drivers, oncogenes, and tumor-suppressor genes, through mining research papers. Figure 6 displays, for each type of cancer, the genes that are not in the driver benchmarks and their respective number of citations found by CancerMine (Query performed on February 2021).

Most genes were cited as cancer genes at least once in the research papers, which suggests even prioritized genes that are not in driver benchmarks can be related to cancer. The remaining non-classified genes should be further evaluated and suggested as possible novel genes for their respective cancer types. Although it is a secondary study, this experiment allows comparing genes known to be drivers with genes not yet known.

To address the biological impact of the findings, we also used the Cancer Genome Interpreter (CGI) datasets to identify drug response and known drivers^32^. The CGI knowledge bases for drug predictions are represented by several publications, the CIVIC database, ASCO and ESMO abstracts, FDA guidelines, among others. The known drivers from CGI datasets represent a complete reference from literature, also including the OncoKB precision oncology knowledge base and ClinVar information about genomic variation. Such analysis showed that genes found by DiSCaGe are associated with drugs and are known drivers, reinforcing the relevance of the findings. The gene sets are presented in the supplementary material.

## Discussion

This work introduced DiSCaGe, a computational method for discovering significant genes for cancer, which takes into account weighted mutations in genes and how they can affect network neighborhood through an asymmetric spreading strength measure. DiSCaGe was built to be easy to use by the end-user, demanding mutation data and gene networks as input in standard formats. Additionally, the user must define mutation weights, with no need to define unclear hyper-parameters, thus facilitating the use. An experimental evaluation was conducted with a set of known cancer genes benchmarks and an automated literature review of genes prioritized by DiSCaGe. DiSCaGe was able to (1) prioritize genes known to be related to cancer, (2) prioritize genes related to cancer with low mutation frequency, (3) suggest genes that are not in benchmarks but are cited in research papers as cancer-related ones, and (4) suggest possible novel cancer genes.

DiSCaGe presents several differences in comparison with related methods. Most methods use the simple frequency or a binary mutation matrix to compute the mutation score, such as MUFFINN^10^, nCOP^11^, and MEMo^9^. DiSCaGe employs a simple way to get weighted frequency based on the definition of weights on the impact of each type o mutation. Such impacts are user-centric, i.e., the final user can define the weights based on the objective of the analysis. DriverML^33^ automatizes the definition of the impact of mutation types through a machine-learning approach, based on previous information. Related to the use of gene interaction networks, nCOP seeks to identify connected subnetworks that are significant altered across the patients, using these finding to ranking the genes, while DiSCaGe does not consider the subnetworks, only the gene neighborhood. In this way, MUFFINN is closely related to DiSCaGe, but the neighborhood influence is obtained through the maximum of the direct neighbor mutation score or the sum of the direct neighbor, divided by its degree. Also, DiSCaGe differs on using the union network to infer the spread strength from a mutated gene, thus considering it on the neighbor influence. Gene interaction networks are the only previous knowledge information that DiSCaGe employs for cancer gene prioritization. None previous information about the gene cancer significance is used. The machine-learning methods use these known cancer genes in order to discover possible novel ones. MutSigCV^31^ is a frequency-based method that estimates the background mutation, while other methods, such as Dendrix^34^ and WExT^35^ uses only mutation data to infer a group of mutually exclusive genes. DiSCaGe does not employ such features.

A limitation of the work refers to a lack of a systematic biological evaluation of the findings. Despite being a complex task, further laboratory in-vitro and in-vivo investigations can be performed to confirm the results of experiments. In this study, it was used an in silico validation using datasets from knowledge bases to assess the relevance of the findings, showing the potential of DiSCaGe to find several genes associated with drugs. However, several other genes could be evaluated as potential novel information, which is beyond the scope of this study. Also, DiSCaGe can be subjected to a pan-cancer study to be evaluated in a large number of cancer types, thus providing subsidies for its characterization and understanding of cases in which it can be appropriately used. Furthermore, DiSCage could allow the analysis of synonymous mutation. A natural extension of DiSCaGe is to allow the method to suggest possible driver pathways with the use of the final network and gene mutation score for finding significantly related genes. Finally, the development of an online tool will facilitate the use of DiSCaGe by end-users.

## Methods

### Step 1: Building the weighted mutation matrix

In this first step, the preprocessed MAF file is used as a source for the construction of the Weighted Mutation Matrix (WMM), in which rows are patients and columns are genes. In WMM matrix *wmm*, entry $wmm_{p_ig_j}$, for each pair of patient $p_i$ in the set of patients *P* and gene $g_j$ in the set of genes *G*, a score is obtained according to its type of mutation *vc* (Variant_Classification from MAF input file) and in a weight *w*(*vc*) assigned for each *vc*. Such weights can be defined by the user in the input of the method according to the purpose of the analysis and the user’s knowledge about the mutation data. However, there is a list of predefined weights in the method, which was predefined based on the functional impact of each mutation related by experts in cancer genomics.

Considering a patient $p_i$, and all mutations in a gene $g_j$, entry $wmm_{p_ig_j}$ is defined as

$$ wmm_{{p_{i} g_{j} }}  = \frac{1}{{\left| {VC_{{p_{i} g_{j} }} } \right|}}\sum\limits_{{vc \in VC_{{p_{i} g_{j} }} }} {w(vc)}  $$

where $VC_{p_ig_j}$ is the list of mutations that patient $p_i$ undergoes in gene $g_j$, and *w*(*vc*) is the weight defined for the type of specific mutation.

Such a process is performed for all patient-gene pairs. Therefore, all pairs of a mutated gene $g_j$ in a patient $p_i$ have a score $wmm_{p_ig_j}$, which represents the importance of that mutation in that patient. The weighted frequency of mutations is used to consider the mutations and the possible functional impact of them and because sometimes it is necessary a set of mutations to initiate the cell carcinogenesis. Furthermore, the use of average avoids possible errors and noise in the sequencing data.

### Step 2: Generating a mutation score for each gene

A single score for each gene is extracted from *wmm*, and called weighted frequency $wf(g_i)$, which is the sum of the gene scores for all patients, divided by the number of patients, defined as

$$\begin{aligned} wf(g_i) = \frac{1}{|P|} \sum _{p_j \in P} wmm_{p_jg_i} \end{aligned}$$

where *P* is the set of patients. Such a frequency is extracted for all genes, generating a set *wf* with weighted frequencies for all genes. The single final score is normalized by the largest value in *wf*, thus yielding a normalized weighted frequency $nwf(g_i)$, defined as

$$\begin{aligned} nwf(g_i) = \frac{wf(g_i)}{\max _{g \in G}(wf(g))} \end{aligned}$$

### Step 3: Consensus of gene interaction networks

An important component of DiSCaGe is the gene network, which significantly impacts the method result. Towards reducing the bias of choice of a single network, DiSCaGe accepts multiple networks as input, i.e., the method can be executed with one or more networks.

Each gene network $GN_i$ of the input set ${GN_1, ..., GN_N}$ was treated as undirected and unweighted networks. The union operation on these networks generates an undirected and weighted network *UGN*. Weights on *UGN* interactions are the average of times an interaction occurs in each network, i.e., the original interactions are maintained, and a bigger weight is assigned to the interactions that appear more times. With this, such interactions would be more likely to exist, once they are in more networks.

### Step 4: Extraction of the Gene Spreading Strength Network (GSSN)

According to the local hypothesis^16^, genes (and their associated proteins) involved in a specific disease tend to interact with each other, and some mutations can influence other genes in the same pathway^36^. In this context, if a gene is mutated, such a mutation can impact its neighbors and propagate to the network.

An adapted spreading strength measure proposed by^37^ was defined for quantifying the spreading strength of a mutated gene through the neighborhood in *UGN*. Such a measure takes into account both direct and indirect neighbors, and quantifies the spread of a mutation from a node $g_i$ to a node $g_j$, defined as

$$\begin{aligned} ss(g_i, g_j) = (1 + r_i \times r_j^{out}) \times p_{ij} \end{aligned}$$

where $r_i$ is the sum of the edge weights of $g_i$; $r_j^{out}$ is the sum of the edge weights of $g_j$ that are not edges of $g_i$, i.e, $r_j^{out} = \sum _{g \in (N(g_j) \setminus N(g_i))} p(g_j, g)$, where *N*(*g*) is the set of neighbors of *g* and $p(g_i, g_j)$ is the weight of edge $(g_i, g_j) \in UGN$; and $p_{ij} = p(g_i, g_j)$. The spreading strength is an asymmetric measure, i.e, $ss(g_i, g_j) \ne ss(g_j, g_i)$. Considering the term $(1 + r_i \times r_j^{out})$, value 1 represents a single spreading from $g_i$ to $g_j$ and $r_i \times r_j^{out}$ denotes the impact of $g_i$ through $g_j$, taking into account their indirect neighbors which are direct neighbors of $g_j$. At the end, such a value is tuned by the weight of the edge $(g_i, g_j)$. The final spreading strength measure is normalized by the largest value of *ss*, thus obtaining a normalized spreading strength $nss(g_i, g_j)$, defined as

$$\begin{aligned} nss(g_i, g_j) = \frac{ss(g_i, g_j)}{\max _{(g, g') \in G \times G}(ss(g, g'))} \end{aligned}$$

After the extraction of the normalized spreading strength measure for all neighbor genes, a directed and weighted network, called Gene Spreading Strength Network (GSSN), is obtained. In GSSN, directed interaction weights represent the degree of spreading at which a mutation in a gene $g_i$ can pass through a gene $g_j$. Finally, the mutation score of each gene is assigned to the GSSN network.

### Step 5: Extraction of mutation neighbors influence

The spreading strength among genes represents how much a single gene can be affected by mutations of its neighborhood, and how much it can affect its neighbors. In this step, the received influence of a mutated gene is extracted by a function $r(g_i)$, which represents how much influence $g_i$ receives from its neighbors, defined as

$$\begin{aligned} r(g_i) = \sum _{g_k \in N(g_i)} nwf(g_k) \times nss(g_k, g_i) \end{aligned}$$

where $N(g_i)$ are direct neighbors of $g_i$ on GSSN. After the calculation of $r(g_i)$ for all genes of *GSSN*, a maximum value normalization is applied on $r(g_i)$, as follows:

$$\begin{aligned} nr(g_i) = \frac{r(g_i)}{\max _{g \in G}(r(g))} \end{aligned}$$

### Step 6: Gene mutation score enrichment based on GSSN and gene prioritization

In this step, the final mutation score of each mutated gene *g* is obtained, taking into account the individual mutation score of gene *nwf*(*g*) and the influence *nr*(*g*) score from its neighbors. The final mutation score $ms(g_i)$ of a gene $g_i$ is the sum of its mutation score and its neighbor’s mutations influence, given by

$$\begin{aligned} ms(g_i) = nwf(g_i) + nr(g_i) \end{aligned}$$

After *ms*(*g*) has been obtained for all genes, the final ranking of prioritized mutated genes is extracted through their sorting by *ms*. Genes with the highest *ms* values are likely to be significantly mutated and related with significantly mutated neighbors.

## Data and preprocessing

### Cancer data

The selected cancer mutation data sets were subjected to a preprocessing routine. This is a crucial activity in cancer data analyses, since such data contain a large amount of information that can be suppressed (intron region, for example) when exome mutation data are analyzed. Furthermore, outlier samples can also be removed from the original data sets. The preprocessing routine involves the following two steps: (1) Maintenance of specific somatic variants: only the following somatic variants were kept in MAF file: 3’UTR, 5’UTR, Frame_Shift_Del, Frame_Shift_Ins, In_Frame_Del, In_Frame_Ins, Missense_Mutation, Nonsense_Mutation, Nonstop_Mutation, Splice_Site, and Translation_Start_Site; and (2) Removal of hypermutated samples: patients are considered hypermutated when they have a much greater number of mutations than most patients in the set. Hypermutated samples should be removed, since they are usually noisy or outliers, which can bias the analyses. Among the several strategies that identify such samples, we used the one proposed by^38^, according to which a sample is hypermutated when it contains more than $(Q3 + 4.5 \times IQR)$ somatic mutations, where *Q*3 is the third quartile, and *IQR* is the interquartile range of the distribution of mutations across all data samples. If a set of hypermutated samples is identified, it is removed from the MAF file.

No specific genes were removed from the data set. For example, FLAGS (frequently mutated genes)^39^ were kept in the analyses, since the aim was the evaluation of the proposed approach with all genes of the preprocessed data set.

### Gene interaction networks

The following two gene networks that use protein-protein interaction (PPIs) as the main source of interactions were selected for the development of the computational approach and the experiments: 1) Reactome Functional Interactions (Reactome); and 2) Human Protein Reference Database (HPRD). Each network was treated as undirected and unweighted network $G = (V, E)$, where set of vertices $V = \{g_1, g_2, ..., g_n\}$ are genes and $(g_i, g_j) \in E$ if gene $g_i$ interacts with gene $g_j$.

Biological networks are known to be incomplete^40,41^. A link prediction approach was used in this research for inferring interactions among genes in a network. According to the local hypothesis^16^, two functionally related genes are likely to share common neighbors^9^. Szymkiewicz–Simpson coefficient ($ssc(g_i, g_j)$)^42^, also known as overlap coefficient, was used for determining how similar two genes $g_i$ and $g_j$ can be, as follows: $ssc(g_i, g_j)~=~{\frac{|N^*(g_i) \cap N^*(g_j)|}{\min (|N^*(g_i)|,|N^*(g_j)|)}}$, where *N*(*g*) is the set of neighbors of *g*, and $N^*(g) = N(g) \cup \{g\}$, which requires the union operator so that the direct link between $g_i$ and $g_j$ can be considered. The overlap coefficient is extracted for each pair of nodes of a gene network *GN*, thus resulting in a new weighted gene network *wGN*, where the weight on the links is the overlap coefficient. A threshold $\gamma$ was defined for keeping the most significant links in *wGN*, in which only edges of a coefficient higher than $\gamma$ are maintained in the network. A similar approach used by^9^ was applied for the choice of an appropriate $\gamma$ threshold, using 186 known pathways derived from KEGG^43,44^ and extracted from MSigBD (database v7.2, updated September 2020)^45,46^. For each pathway *p*, all links among the genes of *p* are selected and the weight is verified in *wGN*. The average overlap coefficient among all links of *p* is calculated, while ten random pathways of same size of *p* are extracted in *wGN*. The link weight average is obtained for each random pathway. As a result, the average overlap coefficient in the network is extracted for each real pathway, considering the known pathway and the set of random pathways, in which, as expected, the known pathways show a higher overlap coefficient. The results of this analysis suggest only links whose overlap coefficient is higher than random choices should be kept in *wGN* for maintaining interactions likely to participate in biological processes. Therefore, the median of values of all random pathways was considered. The thresholds obtained were $\gamma = 0.16$, and $\gamma = 0.20$, for Reactome, and HPRD, respectively. A non-weighted enriched gene network *eGN* was extracted for each network *wGN*, in which the weight of all links in *eGN* is higher than $\gamma$ in the respective *wGN*. The enriched gene networks were called by their original names, with prefix *e* (e.g., the enriched version of HPRD was called eHPRD).

## Supplementary Information

Supplementary Information.
