# Supplementary material for: A computational approach for the discovery of significant cancer genes by weighted mutation and asymmetric spreading strength in networks
Source: Sci Rep. 2021 Dec 7;11:23551. doi: 10.1038/s41598-021-02671-8 (PMC8651746; doi:10.1038/s41598-021-02671-8)
Supplement: Supplementary file 1 — Supplementary Information. [file 41598_2021_2671_MOESM1_ESM.pdf]

A computational approach for the discovery of significant cancer genes by weighted mutation and asymmetric spreading strength in networks

Jorge Francisco Cutigi[1,3,*], Adriane Feijo Evangelista[2], Rui Manuel Reis[2], and Adenilso Simao[3]

[1]Federal Institute of Sao Paulo, Sao Carlos SP, Brazil
[2]Molecular Oncology Research Center – Barretos Cancer Hospital, Barretos SP, Brazil
[3]University of Sao Paulo, Sao Carlos SP, Brazil
[*]cutigi@ifsp.edu.br

ABSTRACT

Identifying significantly mutated genes in cancer is essential for understanding the mechanisms of tumor initiation and progression. This task is a key challenge since large-scale genomic studies have reported an endless number of genes mutated at a shallow frequency. Towards uncovering infrequently mutated genes, gene interaction networks combined with mutation data have been explored. This work proposes DiSCaGe (Discovering Significant Cancer Genes), a computational method for discovering significant genes for cancer. DiSCaGe computes a mutation score for the genes based on the type of mutations they have. The influence received for their neighbors in the network is also considered and obtained through an asymmetric spreading strength applied to a consensus gene network. DiSCaGe produces a ranking of prioritized possible cancer genes. An experimental evaluation with six types of cancer revealed the potential of DiSCaGe for discovering known and possible novel significant cancer genes.

Supplementary material

Algorithms

This supplementary section presents the algorithms of the six steps of DiSCaGe.

Algorithm 1: Step 1 - Building WMM

Data: Mutation data $\mathscr{M}$ in MAF format; Dictionary wc of variant classification weight
Result: Weighted Mutation Matrix (WMM)

$P \leftarrow$ set of patients of $\mathscr{M}$;
$G \leftarrow$ set of genes of $\mathscr{M}$;
$wmm \leftarrow P \times G = \{(p_i, g_j) : p_i \in P \text{ and } g_j \in G\}$;
forall *pairs* $wmm_{p_i g_j} \in wmm$ **do**
  $VC_{p_i g_j} \leftarrow$ list of variant classifications of pair $(p_i, g_j) \in \mathscr{M}$;
  $wmm_{p_i g_j} \leftarrow 0$;
  forall $vc \in VC_{p_i g_j}$ **do**
    $wmm_{p_i g_j} \leftarrow wmm_{p_i g_j} + wc(vc)$;
  end
  $wmm_{p_i g_j} \leftarrow \frac{wmm_{p_i g_j}}{|VC_{p_i g_j}|}$;
end
return wmm;

Algorithm 2: Step 2 - Extracting mutation score for each gene

Data: Weighted Mutation Matrix *wmm*

Result: A set nwf of mutation score for each gene

$P \leftarrow$ set of patients of *wmm*;
$G \leftarrow$ set of genes of *wmm*;
$wf = \{\emptyset\}$;
forall $g_j \in G$ **do**
    $wf_{g_j} \leftarrow 0$;
    forall $p_i \in P$ **do**
        $wf_{g_j} \leftarrow wf_{g_j} + wmm_{p_i g_j}$;
    end
    $wf \leftarrow wf \cup \{wf_{g_j}\}$;
end
$nwf = \{\emptyset\}$;
forall $wf_{g_j} \in wf$ **do**
    $nwf_{g_j} \leftarrow \frac{wf_{g_j}}{\max(wf)}$;
    $nwf \leftarrow nwf \cup \{nwf_{g_j}\}$;
end
return nwf;

Algorithm 3: Step 3 - Building consensus network

Data: A set GN_{SET} of gene networks

Result: A weighted network $UGN(V,E,w)$

$UGN(V,E,w) \leftarrow$ empty graph;
$V \leftarrow$ set of all genes of GN_{SET};
forall $GN(V_{GN},E_{GN}) \in GN_{SET}$ **do**
    forall *interaction* $(g_i,g_j) \in E_{GN}$ **do**
        if $(g_i,g_j) \notin E$ **then**
            $E \leftarrow E \cup \{(g_i,g_j)\}$;
            $w(g_i,g_j) \leftarrow \frac{1}{|GN_{SET}|}$;
        else
            $w(g_i,g_j) \leftarrow w(g_i,g_j) + \frac{1}{|GN_{SET}|}$;
        end
    end
end
return UGN;

Algorithm 4: Step 4 - Building Gene Strength Spreading Network *GSSN*

Data: An weighted network $UGN(V', E', w')$
Result: A directed and weighted network $GSSN(V, E, w)$

$GSSN(V, E, w) \leftarrow$ empty directed and weighted graph;
$V \leftarrow V'$;
$lss = \{\emptyset\}$;
forall *interaction* $(g_i, g_j) \in E_{GN}$ **do**
  $N_{g_i} \leftarrow$ set of neighbors of g_i in UGN;
  $N_{g_j} \leftarrow$ set of neighbors of g_j in UGN;
  $r_{g_i} \leftarrow \sum_g^{N_{g_i}} w'(g_i, g)$;
  $r_{g_j} \leftarrow \sum_g^{N_{g_j}} w'(g_j, g)$;
  $r_{g_i}^{out} \leftarrow \sum_g^{N_{g_i} \backslash N_{g_j}} w'(g_i, g)$;
  $r_{g_j}^{out} \leftarrow \sum_g^{N_{g_j} \backslash N_{g_i}} w'(g_j, g)$;
  $p_{g_i g_j} \leftarrow w'(g_i, g_j)$;
  $ss(g_i, g_j) \leftarrow (1 + r_{g_i} \times r_{g_j}^{out}) \times p_{g_i g_j}$;
  $ss(g_j, g_i) \leftarrow (1 + r_{g_j} \times r_{g_i}^{out}) \times p_{g_i g_j}$;
  $E \leftarrow E \cup (g_i, g_j)$ of weight $w(g_i, g_j) = ss(g_i, g_j)$;
  $lss \leftarrow lss\{ss(g_i, g_j)\}$;
  $E \leftarrow E \cup (g_j, g_i)$ of weight $w(g_j, g_i) = ss(g_j, g_i)$;
  $lss \leftarrow lss\{ss(g_j, g_i)\}$;
end
forall *interaction* $(g_i, g_j) \in E_{GN}$ **do**
  $w(g_i, g_j) \leftarrow \frac{w(g_i, g_j)}{\max(lss)}$
end
return *GSSN*;

Algorithm 5: Step 5 - Extraction of mutation neighbors influence

Data: Mutation score nwf for each gene; A directed and weighted network $GSSN(V, E, w)$
Result: A mutation influence nr received for each gene

$r \leftarrow \{\emptyset\}$;
forall $g_i \in V$ **do**
  $N(g_i) \leftarrow$ neighbors of g_i;
  $r(g_i) \leftarrow 0$;
  forall $g_k \in N(g_i)$ **do**
    $r(g_i) \leftarrow nwf(g_k) \times w(g_k, g_i)$;
  end
  $r \leftarrow r \cup \{r(g_i)\}$;
end
$nr \leftarrow \{\emptyset\}$;
forall $r_{g_i} \in r$ **do**
  $nr_{g_i} \leftarrow \frac{r_{g_i}}{\max(r)}$;
  $nr \leftarrow nr \cup \{nr_{g_i}\}$;
end
return nr;

Algorithm 6: Step 6 - Gene mutation score enrichment

Data: Genes G, Mutation score nwf for each gene; Mutation influence nr received for each gene;
Result: Mutation score ms for all genes

forall $g_i \in G$ **do**
  $ms(g_i) \leftarrow nwf(g_i) + nr(g_i)$;
end
return ms;

Running example

In this section, a running example of DiSCaGe is performed. For this, it is necessary to provide input data and hyper-parameters that are mandatory to run DiSCaGe. For the running example, the MAF file of Table 1 will be considered, and four gene networks illustrated in Figure 1. The required hyper-parameters are presented in Table 2, which shows some defined weights for each variant classification.

Table 1. MAF file for the running example.

Hugo_ Symbol	Chromosome	Variant_ Classification	Reference_ Allele	Tumor_Seq_ Allele2	Tumor_Sample_ Barcode
g_1	7	Missense_Mutation	A	T	p_3
g_1	7	Nonsense_Mutation	G	C	p_3
g_1	7	Frame_Shift_Ins	-	T	p_3
g_1	7	Frame_Shift_Del	C	-	p_3
g_1	7	Translation_Start_Site	A	T	p_3
g_1	7	Missense_Mutation	A	T	p_4
g_2	13	Frame_Shift_Ins	-	C	p_2
g_2	13	In_Frame_Ins	-	TTGTGCTTG	p_2
g_2	13	In_Frame_Del	ATTGG	-	p_2
g_2	13	Nonsense_Mutation	C	T	p_4
g_2	13	3'UTR	G	A	p_4
g_2	13	Missense_Mutation	C	T	p_5
g_3	18	Nonsense_Mutation	C	T	p_2
g_3	18	Frame_Shift_Ins	-	A	p_3
g_3	18	Translation_Start_Site	C	T	p_3
g_3	18	Missense_Mutation	C	T	p_5
g_4	1	Frame_Shift_Del	GC	-	p_3
g_4	1	Translation_Start_Site	C	T	p_3
g_4	1	Missense_Mutation	C	T	p_4
g_4	1	Missense_Mutation	G	A	p_5
g_5	11	Missense_Mutation	T	G	p_2
g_6	12	Nonsense_Mutation	C	G	p_1
g_6	12	Nonstop_Mutation	C	G	p_1
g_6	12	Missense_Mutation	C	G	p_1
g_6	12	Translation_Start_Site	G	A	p_1
g_6	12	In_Frame_Ins	-	GAA	p_3
g_7	17	Nonsense_Mutation	C	T	p_5
g_7	17	Frame_Shift_Ins	-	T	p_3
g_7	17	In_Frame_Ins	-	TTGTGCTTG	p_3
g_7	17	In_Frame_Del	CTGGCT	-	p_3
g_7	17	Frame_Shift_Ins	-	G	p_6
g_7	17	Translation_Start_Site	C	T	p_6
g_8	15	Splice_Site	C	T	p_5
g_8	15	Nonsense_Mutation	C	T	p_5
g_8	15	Frame_Shift_Ins	-	A	p_5
g_{10}	18	Frame_Shift_Del	A	-	p_1
g_{10}	18	Translation_Start_Site	C	T	p_1
g_{10}	18	In_Frame_Ins	-	TAT	p_3
g_{10}	18	Nonsense_Mutation	C	T	p_6
g_{10}	18	3'UTR	A	G	p_6
g_{11}	10	Frame_Shift_Del	A	-	p_2
g_{11}	10	In_Frame_Ins	-	TGTA	p_2
g_{11}	10	In_Frame_Del	CTA	-	p_2

Table 2. Mutation weights.

Variant classification - vc	Weight - $w(vc)$
`Nonsense_Mutation`	1.0
`Missense_Mutation`	0.4
`Splice_Site`	0.4
`Frame_Shift_Del`	1.0
`Frame_Shift_Ins`	1.0
`In_Frame_Del`	0.4
`In_Frame_Ins`	0.4
`3'UTR`	0.2
`5'UTR`	0.4
`Nonstop_Mutation`	0.4
`Translation_Start_Site`	0.2

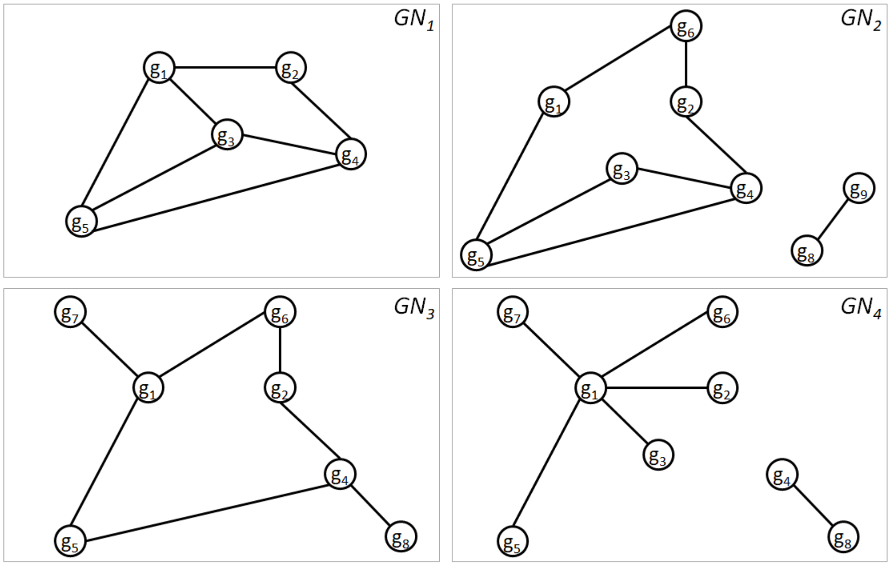

Figure 1. Gene networks for the running example.

Step 1

Considering MAF file from Table 1, patient p_3 has the following list of mutations types in gene g_1: {`Missense_Mutation`, `Nonsense_Mutation`, `Frame_Shif_Ins`, `Frame_Shif_Del`, `Translation_Start_Site`}. As a result, the score $wmm_{p_3,g_1} = \frac{0.4+1.0+1.0+1.0+0.2}{5} = 0.72$. Figure 2 illustrates the WMM for the MAF file of Table 1.

	g_1	g_2	g_3	g_4	g_5	g_6	g_7	g_8	g_{10}	g_{11}
p_1						0.50			0.60	
p_2		0.60	1.00		0.4					0.60
p_3	0.72		0.60	0.60		0.40	0.60		0.40	
p_4	0.40	0.60		0.40						
p_5		0.40	0.40	0.40			1.0	0.80		
p_6							0.6		0.60	

A

Figure 2. An Weighted Mutation Matrix (WMM)

Step 2

Considering gene g_1, the weighted frequency $wf(g_1) = (0+0+0.72+0.4+0+0)/6 = 0.187$. Performing the normalization by the maximum value of the set wf of weighted frequencies, $nwf(g1) = 0.187/0.367 = 0.510$. Table 3 presents the weigthed frequencies wf and normalized weighted frequencies nwf for all genes on the running example.

Table 3. Weighted frequencies.

	wf	nwf
g_1	0.187	0.510
g_2	0.267	0.728
g_3	0.333	0.907
g_4	0.233	0.635
g_5	0.067	0.183
g_6	0.150	0.409
g_7	0.367	1.000
g_8	0.133	0.362
g_{10}	0.267	0.728
g_{11}	0.100	0.272

Step 3

Considering gene networks of Figure 1, the resulting UGN is illustrated in Figure 3. It can be notice that interaction (g_1, g_5) are contained in all individual networks, then such interaction has weight $w((g_1, g_5)) = 1$, while $w((g_1, g_2)) = 0.5$ because interaction (g_1, g_2) is presented in two networks (GN_1 and GN_4).

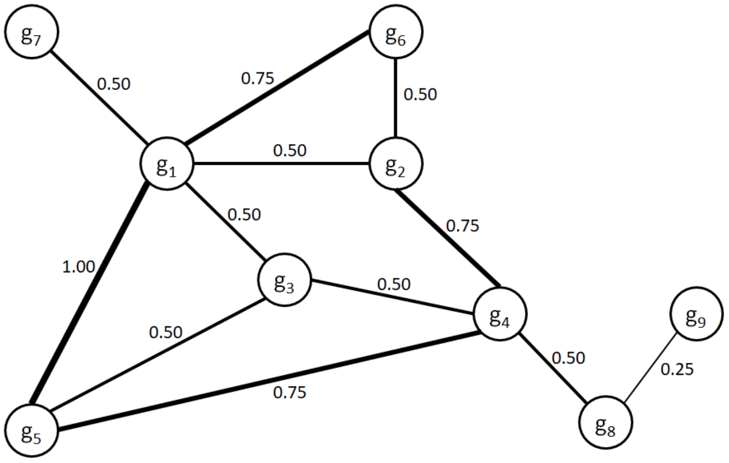

Figure 3. A consensus network UGN, extracted from the union of gene networks of Figure 1

Step 4

Considering UGN of Figure 3 and the strength spreading from g_3 to g_4:

$r_{g_3} = p_{g_3g_1} + p_{g_3g_4} + p_{g_3g_5} = 0.5 + 0.5 + 0.5 = 1.50$

$r^{out}_{g_4} = p_{g_4g_2} + p_{g_4g_8} = 0.75 + 0.5 = 1.25$

$p_{g_3g_4} = 0.50$

$ss(g_3, g_4) = (1 + r_{g_3} \times r^{out}_{g_4}) \times p_{g_3g_4} = (1 + 1.50 \times 1.25) \times 0.50 = 1.438.$

Performing the normalization by the maximum value of the set ss of spreading strength measure, $nss(g1) = 1.438/4.938 = 0.291$.

Figure 4 presents the Gene Strength Spreading Network $GSSN$ generated from UGN of Figure 3.

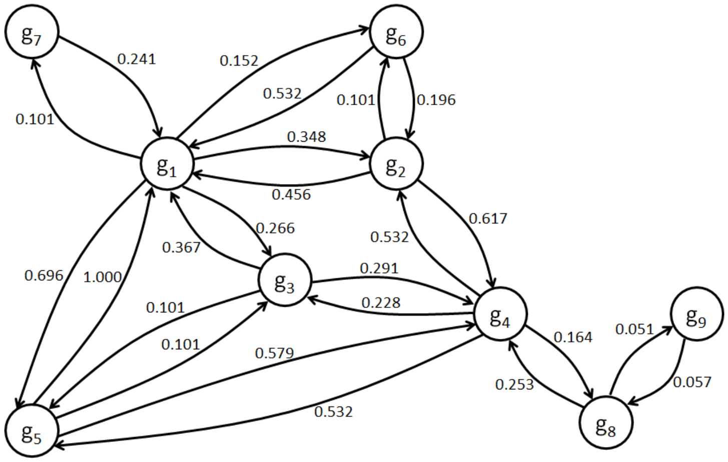

Figure 4. A Gene Strength Spreading Network $GSSN$, extracted from the network of Figure 3

Step 5

Considering gene g_2 and and its neighbors $N(g_2) = \{g_1, g_4, g_6\}$:

$r(g_2) = \sum_{g_k}^{N(g_2)} nwf(g_k) \times nss(g_k, g_2)$

$r(g_2) = nwf(g_1) \times nss(g_1, g_2) + nwf(g_4) \times nss(g_4, g_2) + nwf(g_6) \times nss(g_6, g_2)$

$r(g_2) = 0.510 \times 0.348 + 0.635 \times 0.532 + 0.409 \times 0.196$

$r(g_2) = 0.177 + 0.338 + 0.080$

$r(g_2) = 0.595$

With this, gene g_2 receive from its neighbors a mutation influence score of 0.595. After the calculation of $r(g_i)$ for all genes of *GSSN*, a maximum value normalization is applied on $r(g_i)$, in which $nr(g2) = 0.595/1.306 = 0.455$. Table 4 presents neighbors mutation influence r and normalized influence nr for all genes on the running example.

Table 4. Neighbors mutation influence.

	r	nr
g_1	1.306	1.000
g_2	0.595	0.455
g_3	0.299	0.229
g_4	0.911	0.697
g_5	0.784	0.600
g_6	0.151	0.116
g_7	0.052	0.040
g_8	0.105	0.080
g_9	0.018	0.014
g_{10}	0.000	0.000
g_{11}	0.000	0.000

Step 6

Gene g_2 has own mutation score $nwf(g_2)$ of 0.728, and receives from its neighbors $r(g_2)$ of 0.455. Thus, it results in a final mutation score $ms(g_2) = nwf(g_2) + r(g_2) = 0.728 + 0.455 = 1.183$. Table 5 shows the final mutation score $ms(g_i)$ for every mutated gene g_i of the running example, sorted by $ms(g_i)$.

Table 5. Final mutation score.

gene	$nwf(g_i)$	$r(g_i)$	$ms(g_i)$
g_1	0.510	1.000	1.510
g_4	0.635	0.697	1.332
g_2	0.728	0.455	1.183
g_3	0.907	0.229	1.136
g_7	1.000	0.040	1.040
g_5	0.183	0.600	0.783
g_{10}	0.728	0.000	0.728
g_6	0.409	0.116	0.525
g_8	0.362	0.080	0.442
g_{11}	0.272	0.000	0.272

Cancer Data – additional information

Figure 5 displays the distribution of the mutations before and after the preprocessing task for each cancer data set. It can be noticed that the preprocessed data set the number of mutations is better distributed. For example, regarding PRAD cancer data, in the original data set, the most mutated sample has approximately 10000 mutations, whereas in the preprocessed data set, the most mutated sample had around 100 mutations. The number of mutations, mutated genes, and samples, before and after the preprocessing routine, is also shown in Table 6.

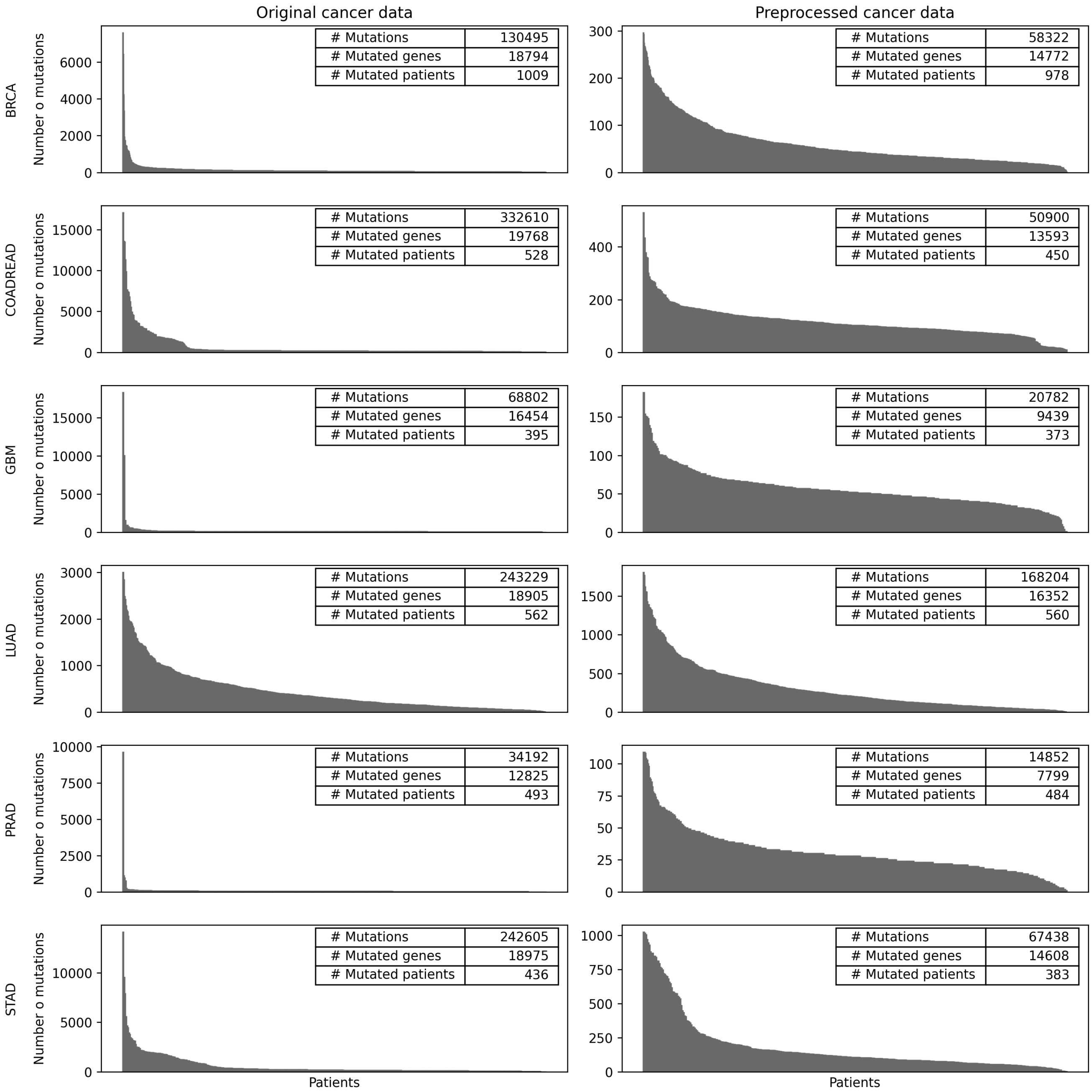

Figure 5. Preprocessing routine: 1) Removing specific somatic mutation variants; and 2) Removal of hypermutated samples.

Table 6. Data set characteristics

	Original data sets			Preprocessed data sets		
**Code**	**Mutations**	**Mutated genes**	**Samples**	**Mutations**	**Mutated genes**	**Samples**
BRCA	130495	18794	1009	58322	14772	978
COADREAD	332610	19768	528	50900	13593	450
GBM	68802	16454	395	20782	9439	373
LUAD	243229	18905	562	168204	16352	560
PRAD	34192	12825	493	14852	7799	484
STAD	242605	18975	436	67438	14608	383

Usage aspects

DiSCaGe allows three types of distinct usage, according to input requirements:

1. The standard usage of DiSCaGe requires mutation data (in MAF format) and one or more gene interaction networks (in a text file with edge list)
2. The MAF files can be exchange by a text file with WMM (Weighted Binary Matrix).
3. In this type of input, the mutation score can be a direct input, not being necessary MAF or WMM files. This way of usage allows the user input a score not necessarily from MAF file, for example, using a input of other tools or method.

In addition to these types of input, the method also allows the definition of different weights for a specific group of genes. In this case, the user can define the weights for all the genes (as already done in the standard method) and also enter a list of genes (for example, known oncogenes), and define different weights for those genes.

Additional results

Precision and DCG

Figure 6 shows a plot with precision for each type of cancer, from the first prioritized genes up to position 200. For example, considering BRCA and a list of 50 prioritized genes, nearly 60% of genes are contained in the driver benchmarks, i.e., they are likely to be a known driver.

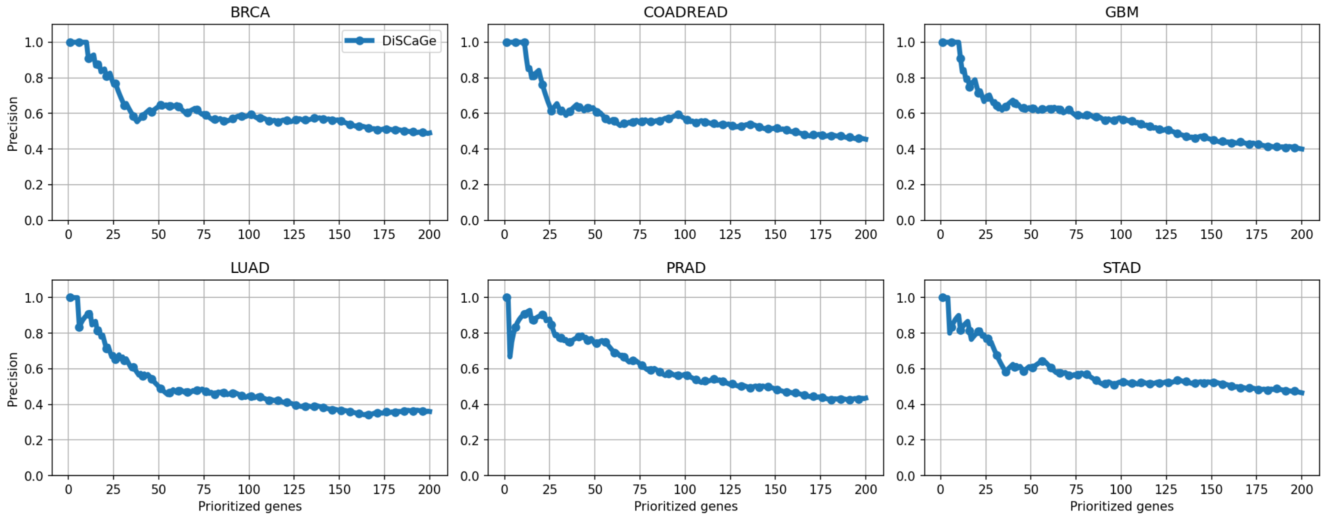

Figure 6. Precision

Figure 7 shows a plot with DCG at a specific position. For example, considering BRCA and a list of 25 prioritized genes, DCG is 40.

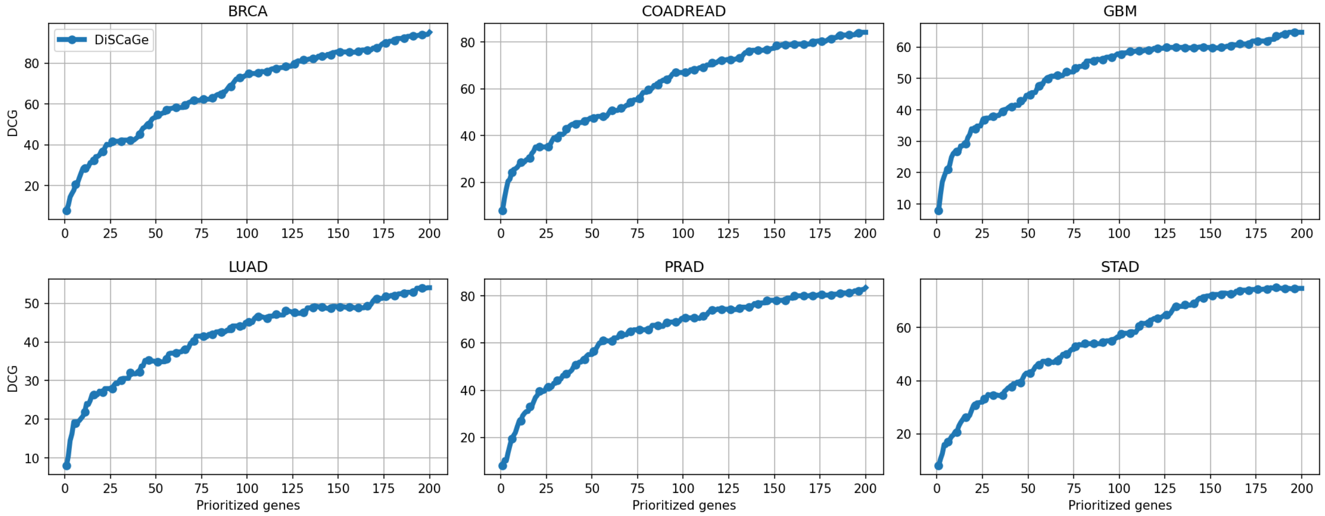

Figure 7. DCG

Comparison with mutation frequency

Not all most frequent mutated genes are considered drivers. Frequency-based methods use complex approaches to estimate background mutation rate to extracted frequencies based on this estimation, which is a challenging task. In this context, the prioritized genes should outperform a ranking of genes sorted by their simple mutation frequency. Figure 8 and 9 presents precision and DCG, respectively, of the results of DiSCaGe in comparison with simple frequency. It can be noticed that DiSCaGe outperforms the results obtained with the simple mutation frequency for all types of cancer.

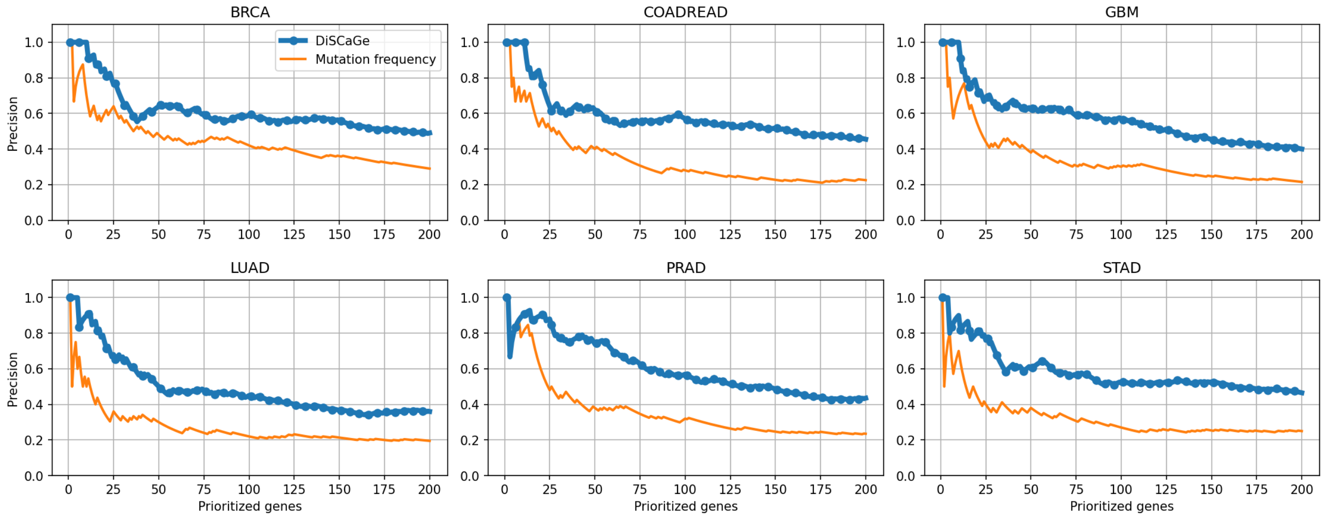

Figure 8. Precision: Method x Frequency

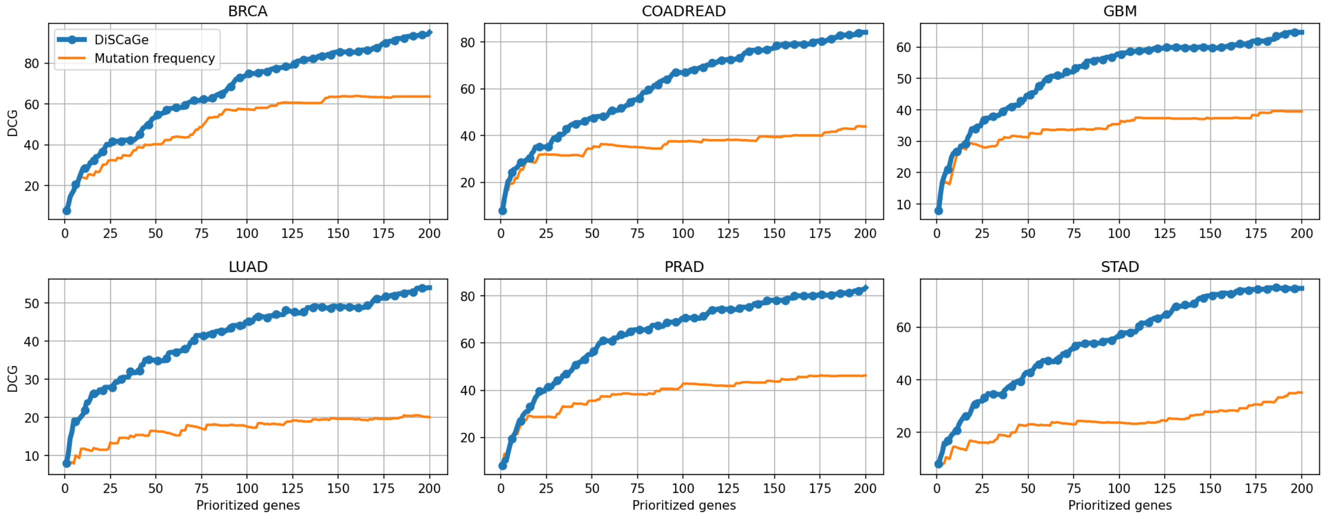

Figure 9. DCG: Method x Frequency

Comparison with weighted frequency

In Step 2 of the DiSCaGe, a mutation score is generated for each gene. Such score is a normalized weighted frequency $nwf(g)$, based on the type of mutations that gene g has on the mutation data. In this experiment, a comparison between the weighted frequency nwf, and the final mutation score ms is performed. Figure 10 and 11 presents precision and DCG, respectively. It can be noticed that the final mutation score obtained by DiSCaGe outperforms the weighted score based only on the gene mutation.

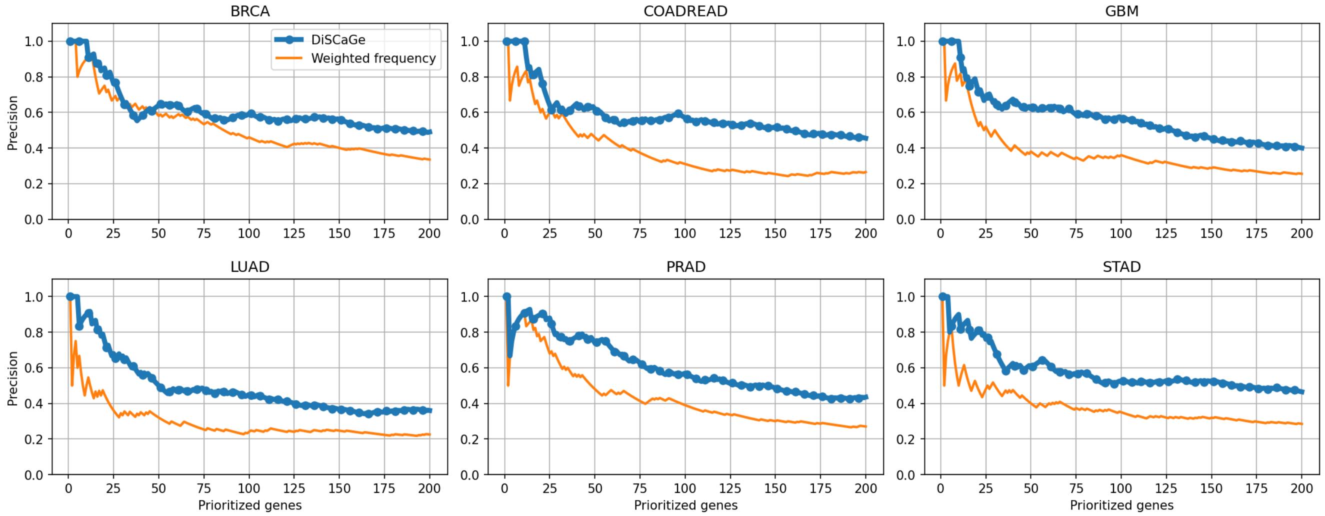

Figure 10. Precision: Method x Weighted Frequency

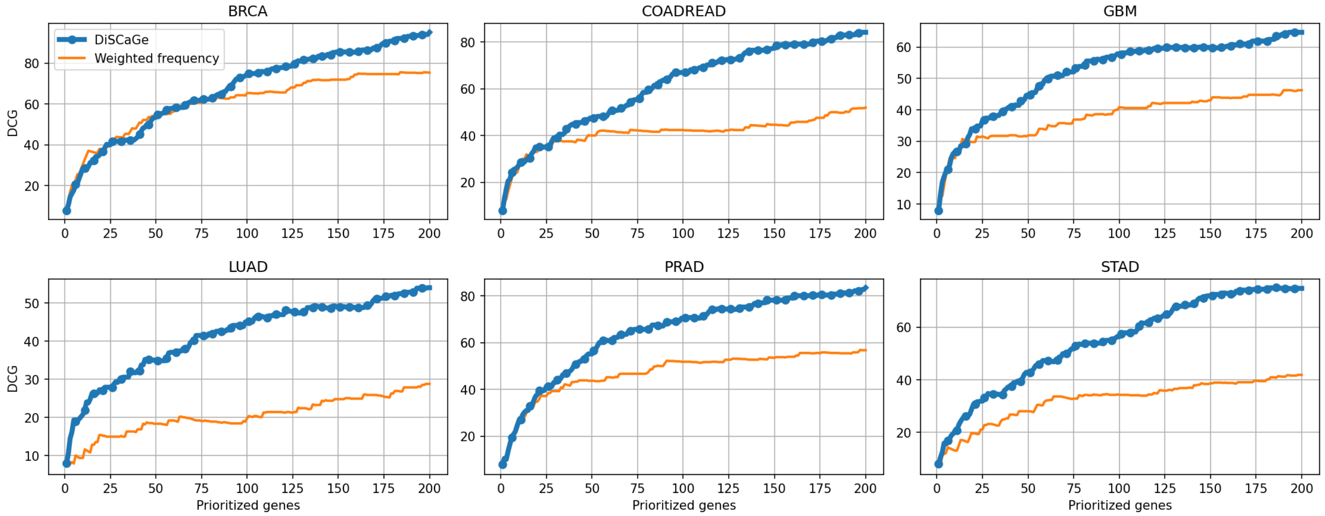

Figure 11. DCG: Method x Weighted Frequency

Comparison with mutation influence from neighbors

Step 4 and Step 5 of the approach extract information to measure the influence of the direct and indirect neighbors on the mutated genes. In this experiment, the neighbors influence $r(g)$ that a gene g receive from its neighbors is compared with the final mutation score $ms(g)$. Figure 12 and 13 presents precision and DCG, respectively. It can be noticed that the precision is similar, considering both scores. However, comparing DCG for the score, it can be noticed that DiSCaGe outperforms the neighbors' influence in all types of cancer, except in LUAD. It suggests that, although the precision is similar, DiSCaGe is likely to return a ranking of genes with high quality. However, it is important to notice that the neighbors' influence has a significant impact on the final result of the method.

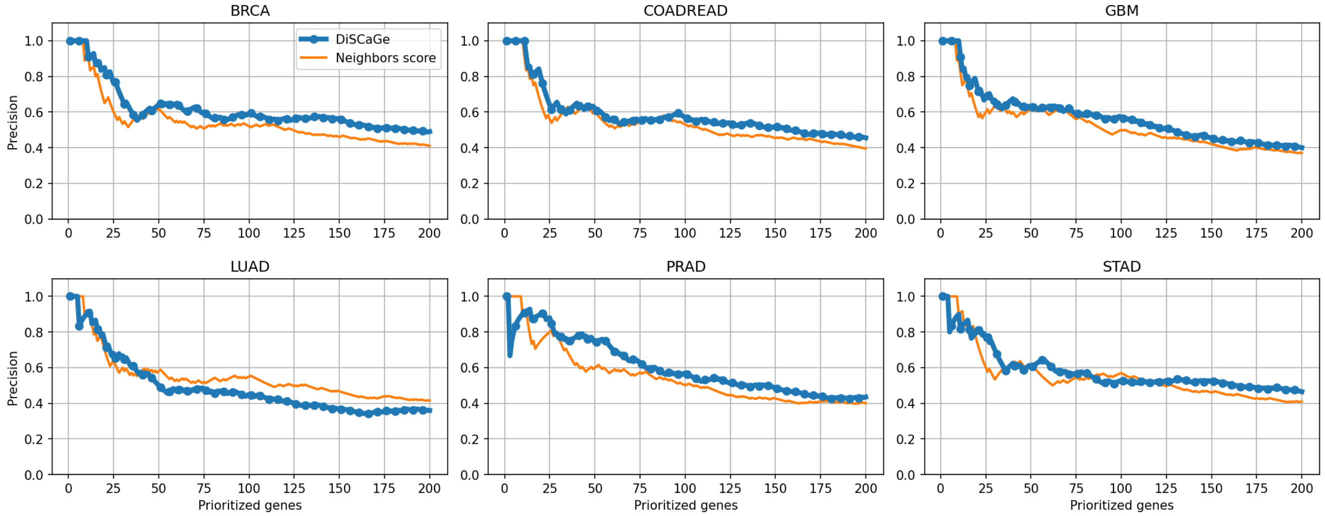

Figure 12. Precision: Method x Neighbors influence

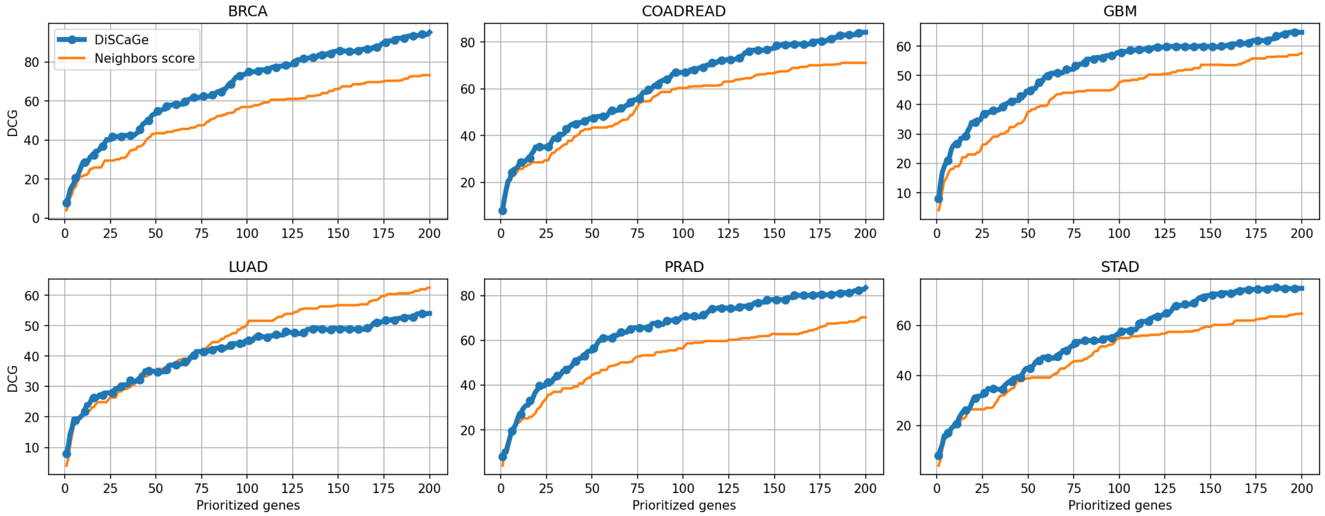

Figure 13. DCG: Method x Neighbors influence

Evidencing the potential of combination of weighted mutation and asymmetric spreading strength

This experiment shows the potential of the combination of the use of weighted mutation and asymmetric spreading strength. For this, A comparison between DiSCaGe and an alternative version was performed. Such an alternative version uses simple mutation frequency for mutation score and value one for the weights of edges on GSSN. As seen in Figure 12 and 13, DiSCaGe outperforms the alternative version on BRCA, GBM, PRAD and STAD. For COADREAD the results are similar and for LUAD the alternative version had better results.

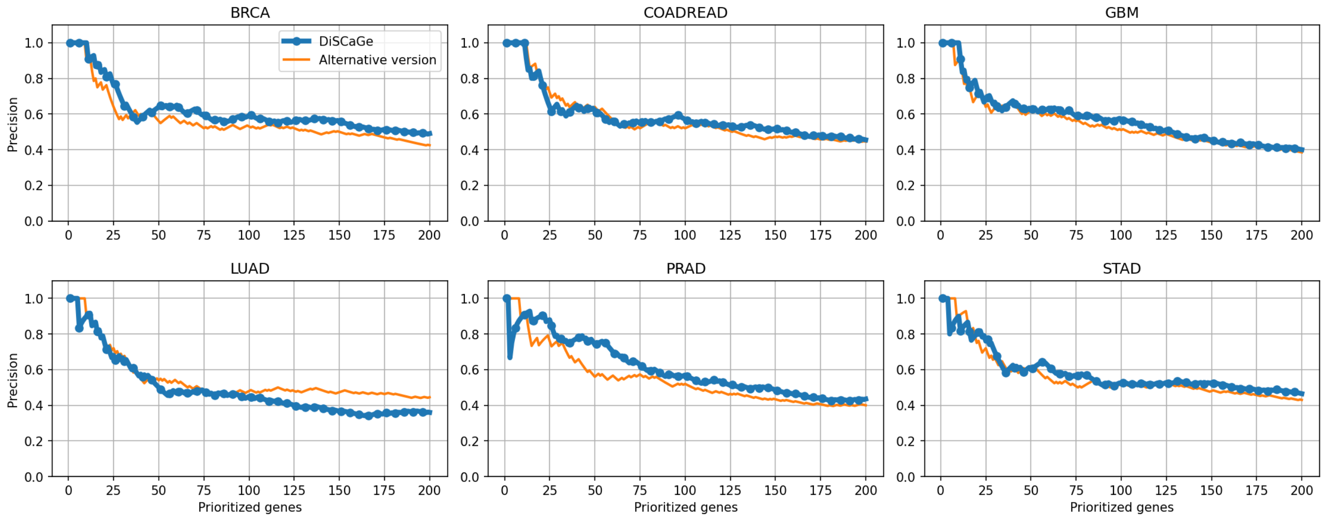

Figure 14. Precision: DiSCaGe x Alternative version

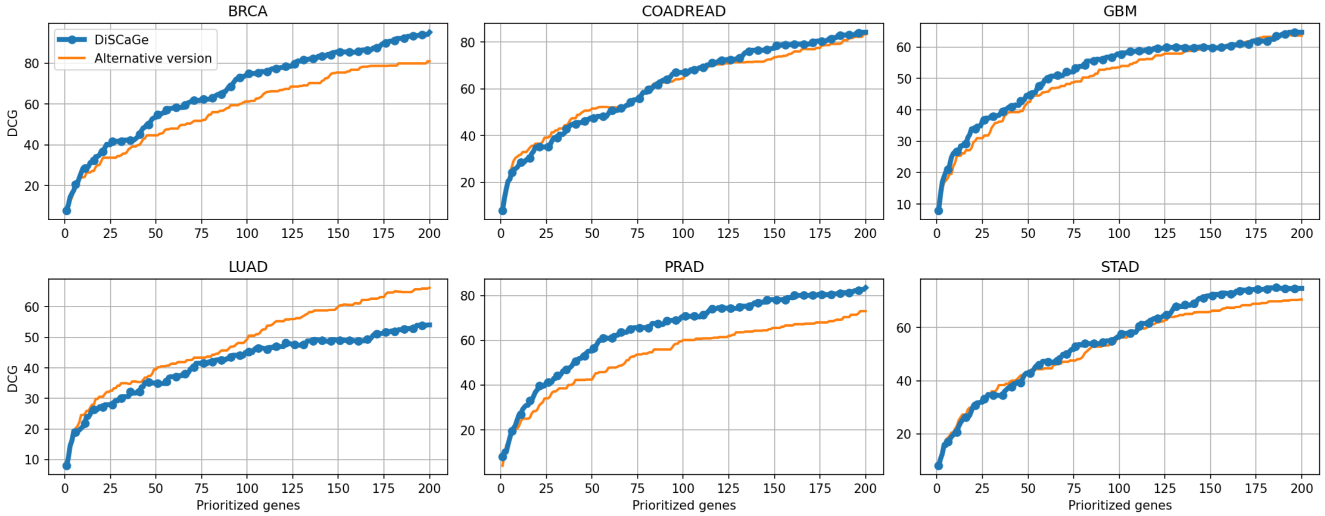

Figure 15. DCG: DiSCaGe x Alternative version

Influence of hyperparameter

The hyperparameter of DiSCaGe is the definition of weights of each type of mutation. Although this hyperparameter is clear and can be defined logically by the expert user, in this experiment an analysis of the influence of the hyperparameter values was performed. For this, the mutation types were divided in three groups, that were defined based on the rationale that mutation of same group have similar functional impact:

Group 1: `Nonsense_Mutation`, `Frame_Shift_Ins`, and `Frame_Shift_Del`.

Group 2: `Missense_Mutation`, `Splice_Site`, `In_Frame_Ins`, `In_Frame_Del`, `5'UTR`, and `Nonstop_Mutation`.

Group 3: `3'UTR` and `Translation_Start_Site`.

In the analysis, a fixed value was defined for two groups, while the other group was variate. The fixed values follow the presented on Table 2, i.e., 1.0, 0.4 and 0.2 for the Groups 1, 2 and 3, respectively. The variation of values was in the range from 0.2 up 1.0.

Figures 16 and 17 show the precision and DCG, respectively, for the variation of the weights of mutation of Group 1. The boxplots represent all values of precision and DCG for the top 200 prioritized genes for each type of cancer. In a general way, it can be noticed that the DiSCaGe performance increases when the values of the weights are higher. For COADREAD and PRAD this evidenced not so clear after value 0.6.

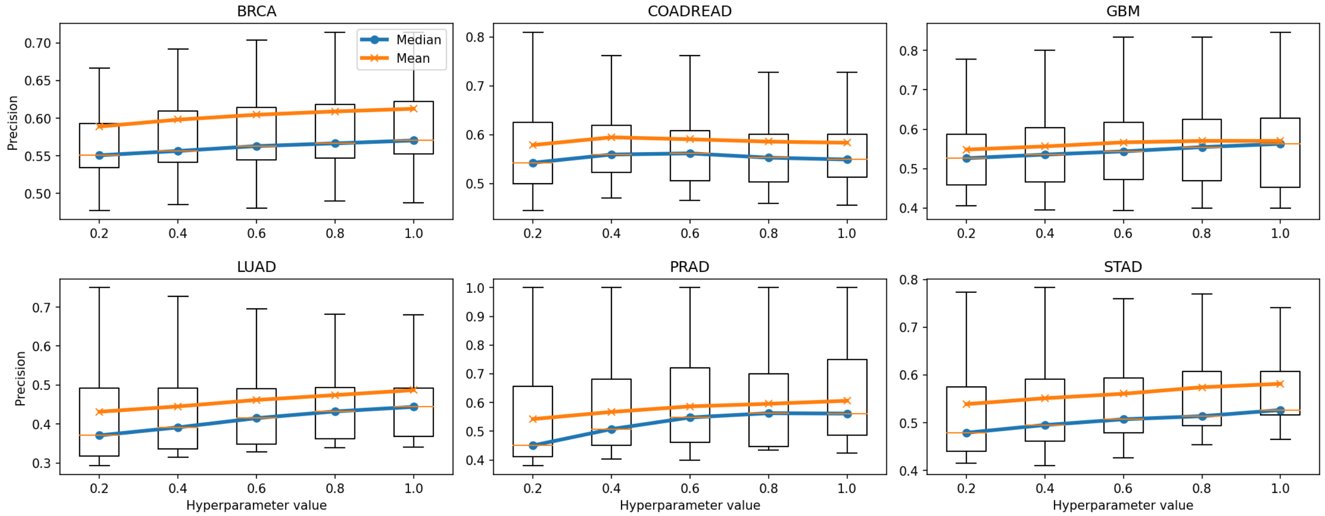

Figure 16. Precision considering the variation on the weights of mutations of Group 1

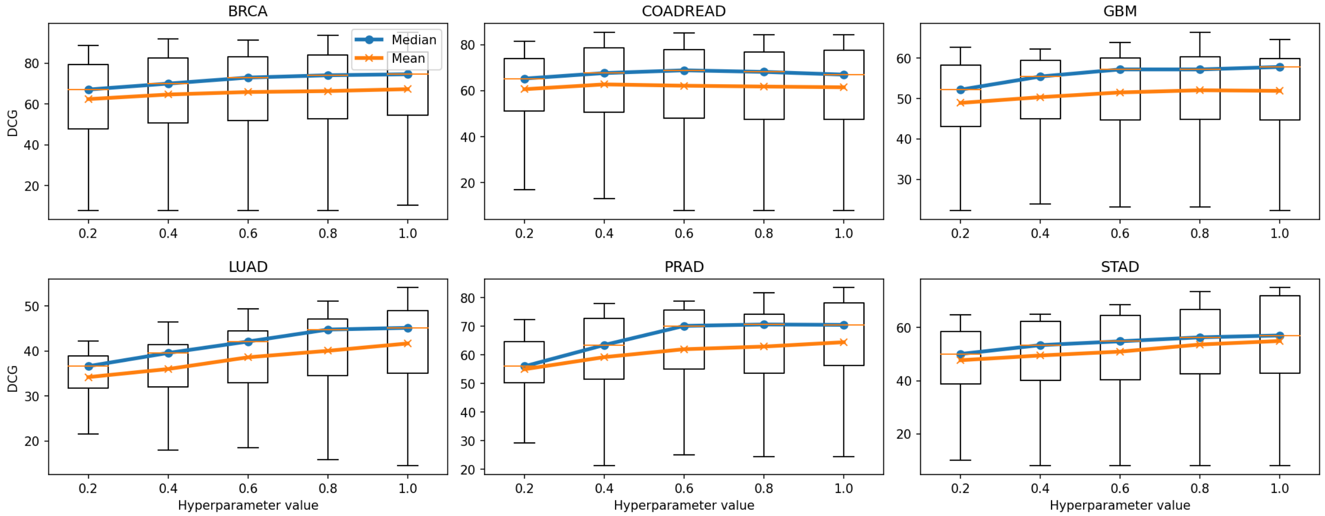

Figure 17. DCG considering the variation on the weights of mutations of Group 1

Related to the weights for the mutation of Group 2, Figures 18 and 18 show the precision and DCG, respectively. It can be noticed that there is no a clear tendency for all cancer types. For example, for LUAD and STAD, the value 0.2 has a better performance. This lack of a clear tendency can be related to the number of missense mutations, which are the majority of the mutations. LUAD is a hypermutated cancer type, and has a large number of missense that can be with no impact, then a lower weight for missense can improve results for LUAD.

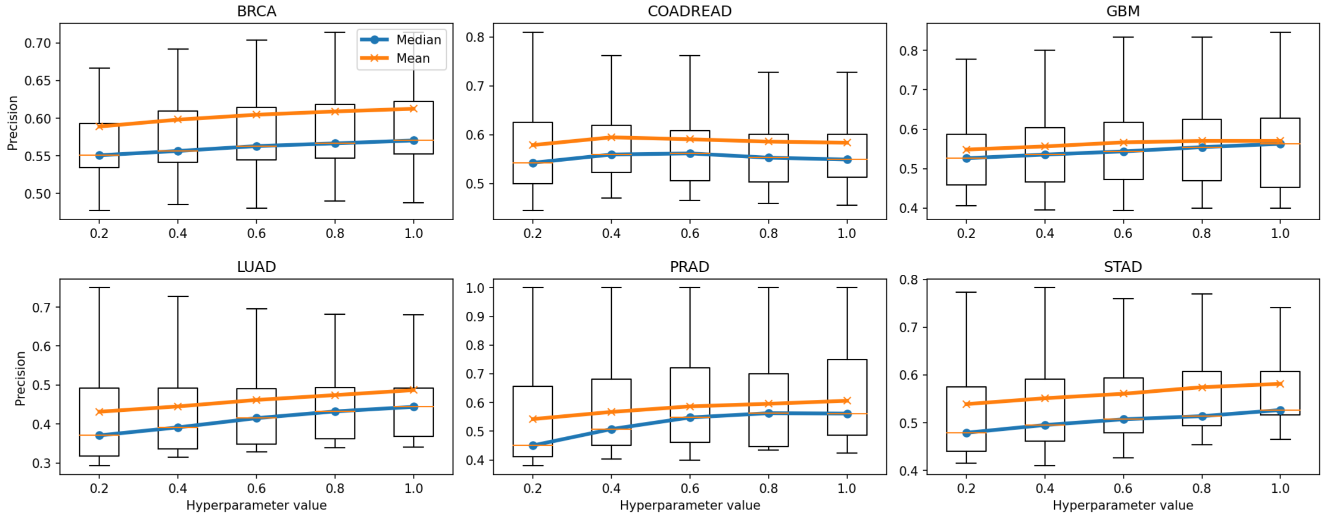

Figure 18. Precision considering the variation on the weights of mutations of Group 2

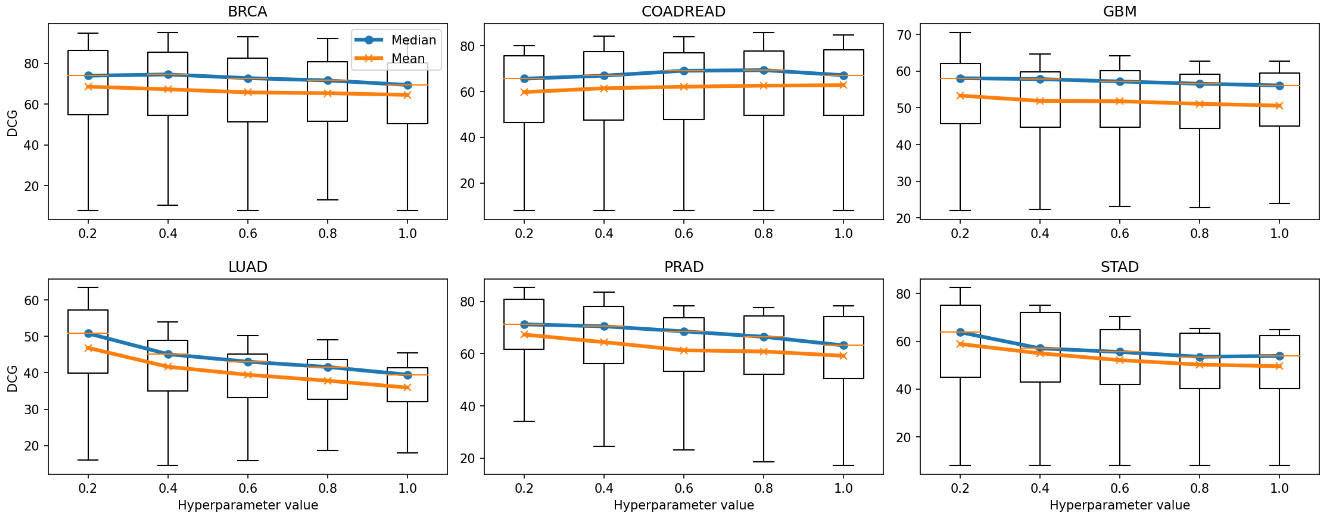

Figure 19. DCG considering the variation on the weights of mutations of Group 2

For the mutation types o Group 3, the performance of DiSCaGe have no significant difference, as shown in Figures 20 and 21. The cause of this behavior can be due to the number of mutations for the types of Group 3 in small, then the impact is less significant.

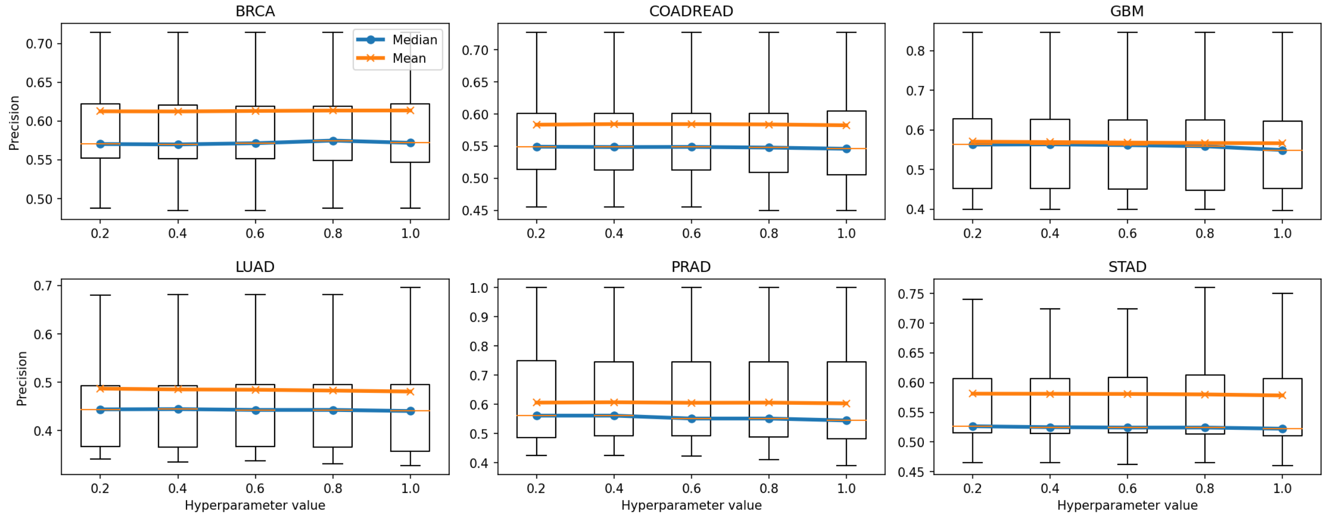

Figure 20. Precision considering the variation on the weights of mutations of Group 3

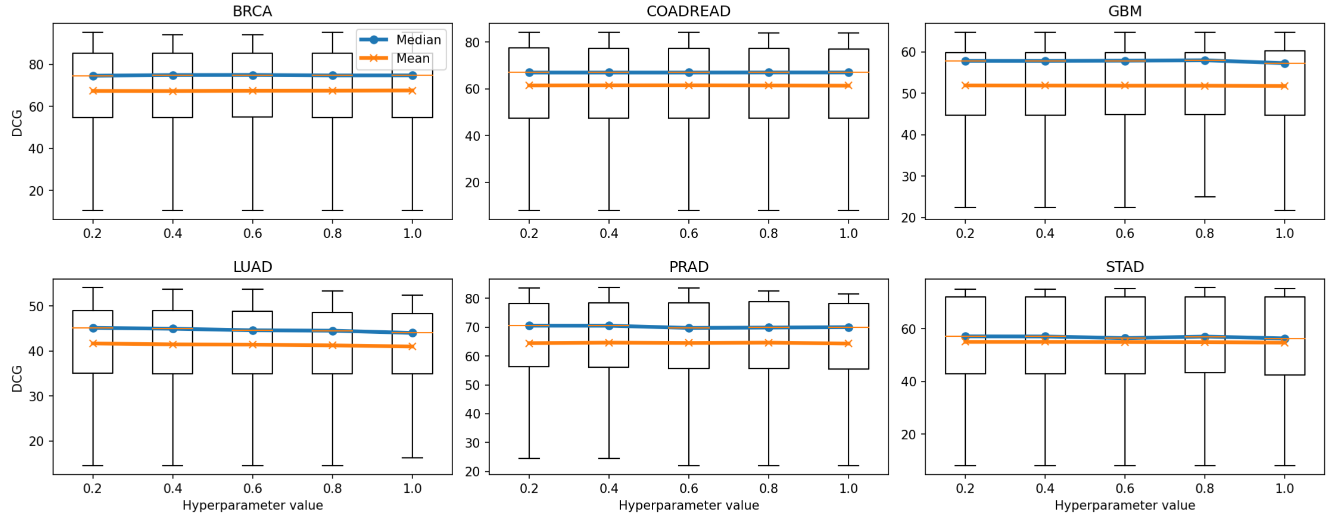

Figure 21. DCG considering the variation on the weights of mutations of Group 3

Additional comparison to related methods

Low-frequency mutated genes

DiSCaGe is able to suggest possible cancer genes with very low mutation frequency. In order to compare this ability with previous methods, Figure 22 shows boxplots of mutation frequency of top 200 genes prioritized for DiSCaGe and related methods. The mutation frequency median is lower for COADREAD and similar to MUFFINN for BRCA and STAD. It is important to mention that this analysis is quite relative, because the fact of finding more genes with low frequency is good or bad is dependable on the objective of analysis.

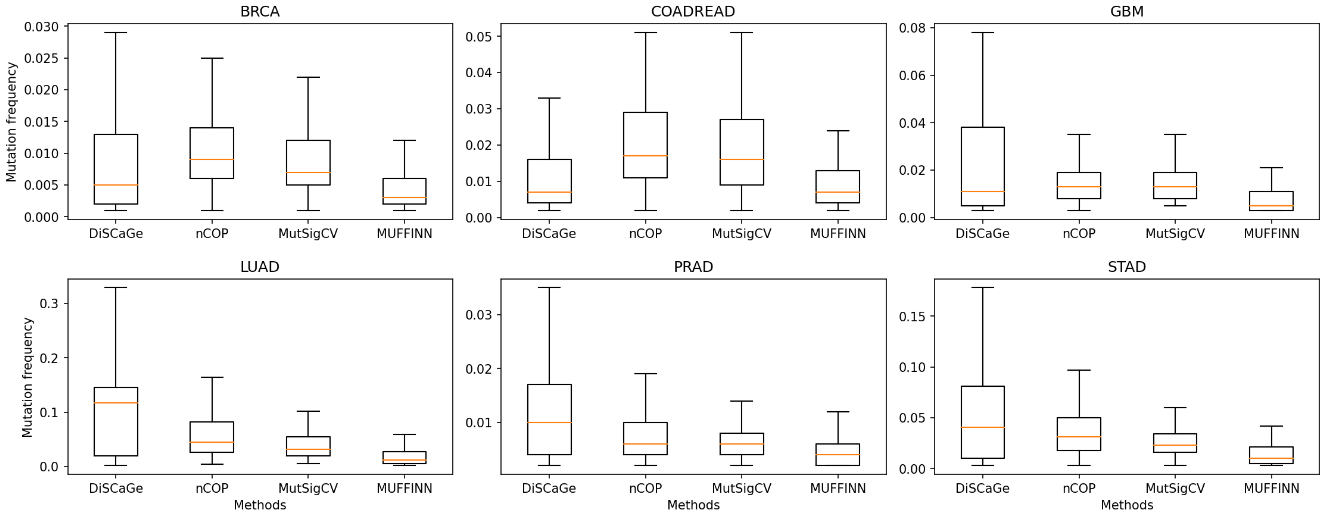

Figure 22. Boxplots of mutation frequencies of prioritized genes

Potential on discovering possible novel cancer genes

Figure 23 shows stacked bar plots of the frequency of top 200 genes prioritized by DiSCaGe and related methods appears in driver benchmarks (green bar), false-positive benchmark (red bar), in CancerMine cited at least one time (blue bar), and not appearance in any of them (gray bar). It can be noticed that MUFFINN prioritized most known cancer-related genes, including evidences from CancerMine, but DiSCaGe outperforms MUFFINN on finding genes on driver benchmarks. DiSCaGe finds a significant number of false-positives for LUAD, PRAD and STAD, but DiSCaGe was the better of finding genes in driver benchmarks in such cancer types.

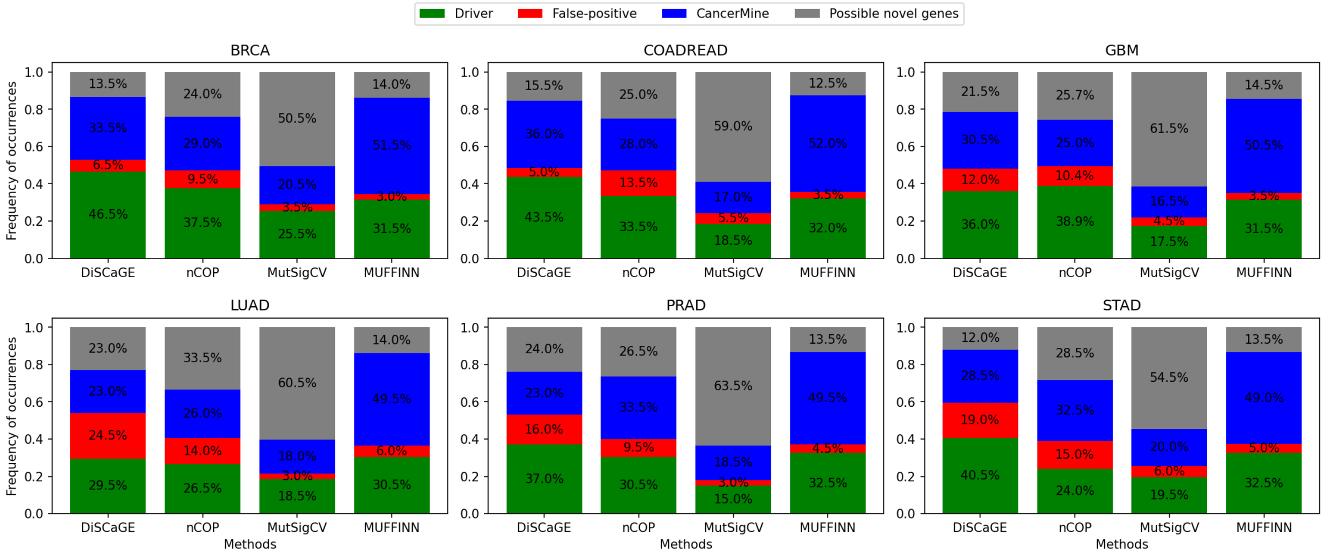

Figure 23. Frequency of occurrences of prioritized genes on benchmarks and CancerMine

Genes associated with drugs and known drivers

An additional *in silico* validation step was performed using the datasets from Cancer Genome Interpreter (CGI). Table 7 presents the top 30 genes prioritized by DiSCaGe that are found in CGI (drugs and known drivers) in the alterations from data used as input for all the analyses in this study.

Table 7. Analyses from Cancer Genome Interpreter (CGI).

BRCA		COADREAD		GBM		LUAD		PRAD		STAD	
**drugs**	**known driver**	**drugs**	**known driver**	**drugs**	**known driver**	**drugs**	**known driver**	**drugs**	**known driver**	**drugs**	**known driver**
AKT1	AKT1	AKT1	AKT1	ABL1	CREBBP	AKT1	AKT1	AKT1	AKT1	AKT1	BRCA1
AR	BRCA1	APC	APC	EGFR	EGFR	AR	CTNNB1	APC	APC	AR	CTNNB1
BRCA1	CDH1	AR	CREBBP	ESR1	NF1	BRCA1	EGFR	ATM	ATM	ARID1A	PIK3CA
CDH1	CREBBP	BRCA1	CTNNB1	MAPK1	PIK3CA	CTNNB1	KRAS	CTNNB1	CTNNB1	BRCA1	SMAD4
EGFR	EGFR	CTNNB1	KRAS	NF1	PIK3R1	EGFR	STAT3	EGFR	FOXA1	CTNNB1	STAT3
ESR1	ESR1	EGFR	PIK3CA	PIK3CA	PTEN	ESR1	TP53	ESR1	PIK3CA	EGFR	TP53
GATA3	PIK3CA	ESR1	SMAD4	PIK3R1	RB1	KRAS		FOXA1	PTEN	ESR1	
MAPK1	STAT3	KRAS	TP53	PTEN	TP53	LRP1B		PIK3CA	SPOP	LRP1B	
PIK3CA	TP53	PIK3CA		RB1		MAPK1		PTEN	TP53	MAPK1	
PIK3R1		PIK3R1		TP53		PIK3R1		TP53		PIK3CA	
TP53		SMAD4				TP53				PIK3R1	
		TP53								SMAD4	
										TP53	
